# A selective inhibitor reveals roles for LONP1 in mitochondrial proteostasis and OXPHOS dependencies

**DOI:** 10.26508/lsa.202503535

**Published:** 2026-10-01

**Authors:** Zsolt Szilagyi, Pauline Michon, Carlos Pardo-Hernández, Mãdãlina Sãcultanu, Javier Miralles Fusté, Andrew M Griffin, Paul S Charifson, Cindy Phan, Yonghong Shi, Gunther Kern, Gabriel Martinez Botella, Thomas A Keating, Bertil Macao, Nils-Göran Larsson, Maria Falkenberg, Jeremy Green, Claes M Gustafsson

**Affiliations:** 1 Department of Medical Biochemistry and Cell Biology, University of Gothenburg , Gothenburg, Sweden; 2 Division of Molecular Metabolism, Department of Medical Biochemistry and Biophysics, Karolinska Institutet , Stockholm, Sweden; 3 Pretzel Therapeutics, Mölndal, Sweden; 4 Pretzel Therapeutics, Waltham, MA, USA

## Abstract

Lon protease 1 (LONP1) is a conserved hexameric protease implicated in mitochondrial disorders and cancer progression. In this study, we present PZL-26, a potent and selective small-molecule inhibitor that targets LONP1 without affecting the proteasome, leading to selective accumulation of mitochondrial proteins. Using PZL-26 in a whole-genome CRISPR-Cas9 screen, we identified genes essential for cell survival under protease inhibition, supporting a role for LONP1 in key mitochondrial processes, including complex I biogenesis, mitochondrial transcription, and translation. Our CRISPR screen results are consistent with proteomics analysis, with both approaches converging on the same mitochondrial pathways and highlighting functional interactions between LONP1 and other mitochondrial proteases, including potential compensatory mechanisms. These findings establish PZL-26 as an effective tool for exploring LONP1 function and pave the way for future therapeutic strategies targeting LONP1 in mitochondrial diseases and cancer.

## Abstract (precis)

PZL-26 targets LONP1 with nanomolar potency and on-target cellular activity, revealing OXPHOS and mitochondrial gene expression as key survival dependencies.

## Introduction

Mitochondrial proteostasis is critical for cellular function and is maintained in part by LONP1, an ATP-dependent serine protease located in the mitochondrial matrix. LONP1 selectively recognizes and degrades oxidatively damaged, misfolded, or superfluous proteins, thereby preventing their accumulation and ensuring mitochondrial protein quality control. Human LONP1, a member of the AAA+ family of ATPases, comprises a Lon-associated N-terminal domain, an AAA+ domain for ATP binding and hydrolysis, and a protease domain. Six LONP1 monomers assemble into a barrel-shaped hexameric structure, with the Lon-associated N-terminal domain interacting with fully or partially unfolded substrates. ATP hydrolysis facilitates substrate threading into the proteolytic chamber, where degradation occurs via a serine–lysine dyad at the active site of the protease domain (Szczepanowska & Trifunovic, 2022).

The essential nature of LONP1 is underscored by the lethality of its deletion in mice and its association with mitochondrial diseases (Quiros et al, 2014). Pathogenic *LONP1* mutations, particularly those affecting the ATP-binding pocket, impair ATP hydrolysis, leading to reduced respiratory chain capacity. *LONP1* mutations have been linked to conditions such as the cerebral, ocular, dental, auricular, and skeletal syndrome (Dikoglu et al, 2015; Strauss et al, 2015), pyruvate dehydrogenase deficiency (Nimmo et al, 2019), and classical mitochondrial disease with oxidative phosphorylation (OXPHOS) deficiency and mitochondrial DNA (mtDNA) depletion (Peter et al, 2018).

LONP1 has been proposed to play a complex role in regulating mitochondrial transcription factor A (TFAM) and, consequently, mtDNA levels. In *Drosophila*, LONP1 has been shown to directly regulate TFAM levels and mtDNA content (Matsushima et al, 2010), but in human cells, this relationship appears to be more intricate. Previous studies suggest that LONP1 preferentially degrades DNA-free TFAM (Lu et al, 2013). Perturbation of LONP1 activity has been associated with altered TFAM and mtDNA homeostasis, although the relationship between LONP1-dependent TFAM turnover and mtDNA levels appears to be complex (Peter et al, 2018; De Gaetano et al, 2020). DNA-free TFAM may arise through several mechanisms, including mtDNA depletion or impaired intrinsic HMG-box DNA-binding activity (Lu et al, 2013). Phosphorylation within HMG box 1 has also been proposed to reduce TFAM-DNA binding and thereby increase its susceptibility to LONP1-dependent degradation (Lu et al, 2013). LONP1-dependent turnover of DNA-free TFAM could therefore contribute both to mitochondrial protein quality control and to regulated fine-tuning of TFAM abundance and function. Inactivating mutations in the two HMG boxes of TFAM (HMG1 and HMG2) that disrupt DNA binding likewise promote LONP1-dependent degradation, whereas phosphomimetic substitutions do so only within HMG1, not HMG2 (Lu et al, 2013).

Emerging research highlights LONP1 as a promising target for cancer therapy because of its crucial role in maintaining mitochondrial homeostasis and promoting tumor cell survival (Shetty et al, 2024). LONP1 is frequently up-regulated in various cancers, where it supports tumor growth by preserving mitochondrial function and protecting cells from apoptosis (Quiros et al, 2014; Sung et al, 2018). Its functions extend beyond protein degradation, as LONP1 also plays a chaperone-like role, enhancing the stability of key mitochondrial components under oncogenic stress (Gibellini et al, 2020). Inhibition of LONP1 disrupts mitochondrial proteostasis, leading to the accumulation of dysfunctional mitochondria, oxidative stress, and, ultimately, cancer cell death (Lee et al, 2021). However, despite its recognized importance, the specific proteolytic targets of LONP1 remain incompletely characterized, necessitating the development of advanced investigative tools to fully understand its therapeutic potential (Shetty et al, 2024).

In this study, we report the discovery of novel, potent LONP1 inhibitors with improved drug-like characteristics for investigating LONP1 biology in vitro. These inhibitors provide a critical tool for probing LONP1 function, enabling exploration of its cellular effects and direct targets. Our findings lay the groundwork for future investigations into LONP1’s roles in mitochondrial dynamics, stress responses, mitochondrial gene expression, and disease processes, deepening our understanding of its physiological significance and therapeutic potential.

## Results

### Optimization of LONP1-selective inhibitors

**Figure 1.**
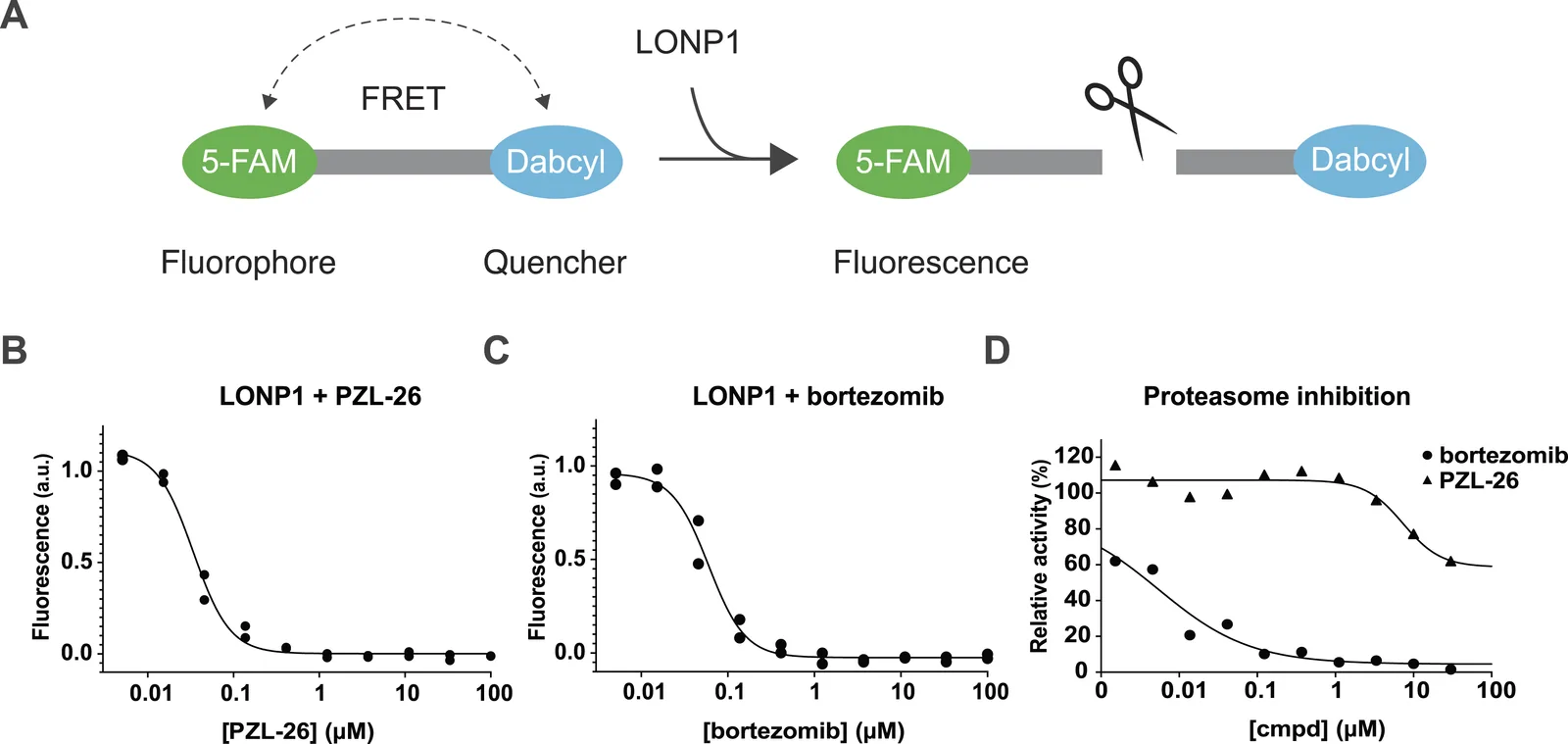
Bortezomib and compound PZL-26 inhibit LONP1 activity using a short peptide substrate. **(A)** Schematic presentation of the FRET-based protease assay, using a peptide substrate to monitor LONP1 and 20S proteasome activity in vitro. **(B)** LONP1 inhibition curve using a short peptide substrate with PZL-26. The calculated IC_50_ value is ∼27 nM. **(C)** LONP1 inhibition curve using a short peptide substrate with bortezomib. The calculated IC_50_ value is ∼40 nM. **(D)** Comparative analysis of the inhibitory effects of bortezomib and PZL-26 on the 20S proteasome. Source data are available for this figure.

Building on the structural insights of Kingsley and colleagues, we employed a structure-guided approach to identify potent and selective LONP1 inhibitors (Kingsley et al, 2021). From a set of boronic acid–based analogs, we identified PZL-26 as a lead compound. A LONP1 activity assay using a short FRET peptide substrate was employed to evaluate compounds from this series. The assay measures changes in fluorescence intensity resulting from peptide hydrolysis, which correlates with LONP1’s enzymatic activity (Fig 1A). PZL-26 was identified as a potent inhibitor with an IC_50_ of 27 nM (Fig 1B). Interestingly, bortezomib, which has previously been used to inhibit LONP1, demonstrated similar potency, inhibiting LONP1 with an IC_50_ of 40 nM (Fig 1C). Unlike bortezomib, however, PZL-26 is selective for LONP1, showing very low activity against the 20S proteasome. Although bortezomib inhibited the 20S proteasome with an IC_50_ of 5.4 nM, PZL-26 was ∼1,000-fold less potent, with an IC_50_ of 7.3 μM (Fig 1D). These findings are in line with the activity profiles reported by Kingsley et al (2021) and further support the approach of stereochemical modification to achieve LONP1 selectivity.

**Figure 2.**
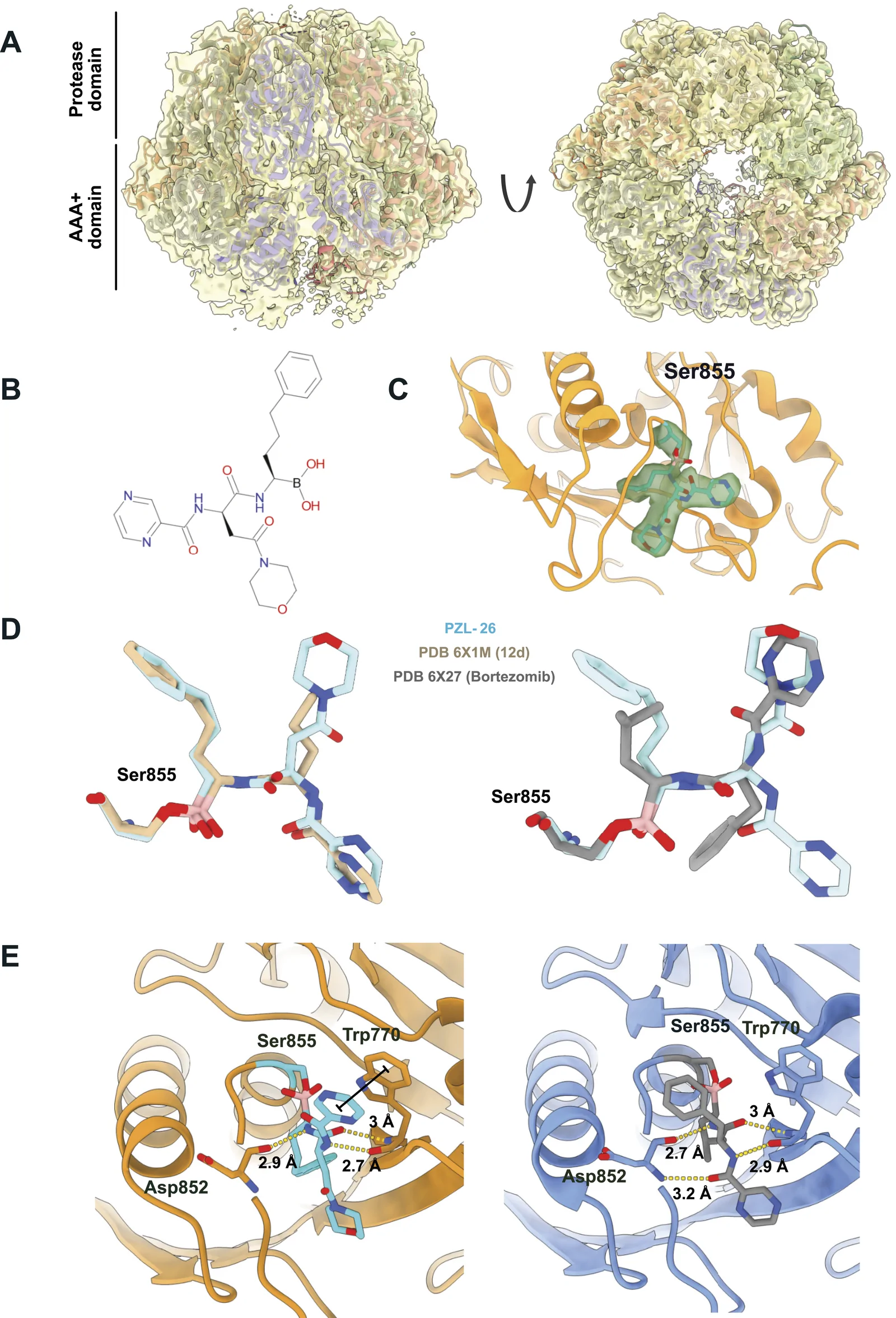
Cryo-EM structure of PZL-26 bound to LONP1. **(A)** Surface representation of the human LONP1 hexamer. Two orientations of the cryo-EM structure are shown, with the AAA+ domain and protease domain labeled. The architecture reveals the hexameric barrel structure with a central pore, characteristic of ATP-dependent proteases (PDB 31SY). **(B)** Chemical structure of PZL-26, highlighting the boronic acid moiety. **(C)** PZL-26 bound in the catalytic pocket of LONP1, with the ligand fitted into the cryo-EM electron density map. The covalent bond between Ser855 and the boronic acid is shown clearly. **(D)** Structural overlay of PZL-26 with two reference inhibitors: compound 12d (PDB 6X1M, left) and bortezomib (PDB 6X27, right). **(E)** Close-up view of the binding pocket of PZL-26 (left) and bortezomib (right, PDB 6X27) in LONP1, showing key interactions. π-stacking and hydrogen bond interactions of PZL-26 with Trp770 are detailed, with measured distances between interacting residues highlighted in yellow in the figure.

To understand the basis for PZL-26’s selectivity, we determined the structure of LONP1 bound to PZL-26 (PDB 31SY) using single-particle cryo-electron microscopy (cryo-EM) (Fig 2A). The chemical structure of PZL-26 is shown in Fig 2B. In the 3 Å structure, we identified a well-defined density in the catalytic site of LONP1, allowing us to unambiguously fit PZL-26 (Fig 2C), where the catalytic dyad Ser855-Lys898 facilitates hydrolysis of incoming threaded substrates (Gesé et al, 2021 *Preprint*; Shin et al, 2021; Mindrebo & Lander, 2025). The boronic acid group of PZL-26 forms a covalent bond with the reactive oxygen of Ser855, as proposed for other covalent inhibitors like bortezomib (Maneix et al, 2021; Shin et al, 2021). This interaction blocks substrate binding and hydrolysis within the proteolytic chamber, ensuring stable inhibition over time.

In prior work, Kingsley et al used a high-throughput screening strategy followed by structure–activity relationship analysis to develop a series of bortezomib analogs, culminating in the identification of compound 12d (PDB 6X1M) with enhanced selectivity for LONP1 (Kingsley et al, 2021). In their study, stereochemical inversion at the P2 position of the inhibitor was shown to induce a distinct reorientation of the P2 and P3 substituents, with the pyrazine amide now occupying the S2 region and the alkyl substituent reoriented into the S3 subpocket, thus improving selectivity for LONP1 over the 20S proteasome. Based on these structural insights, we hypothesized that extending the P2 alkyl substituent to better mimic the pyrazine amide might enhance LONP1 potency while retaining proteasome selectivity, ultimately leading to PZL-26. Structural overlay of compound 12d with PZL-26 in the covalently bound form (Fig 2D, left) reveals a high degree of similarity in stereochemistry and binding conformation, in contrast to bortezomib (Fig 2D, right), which adopts a distinct binding mode.

**Figure S1.**
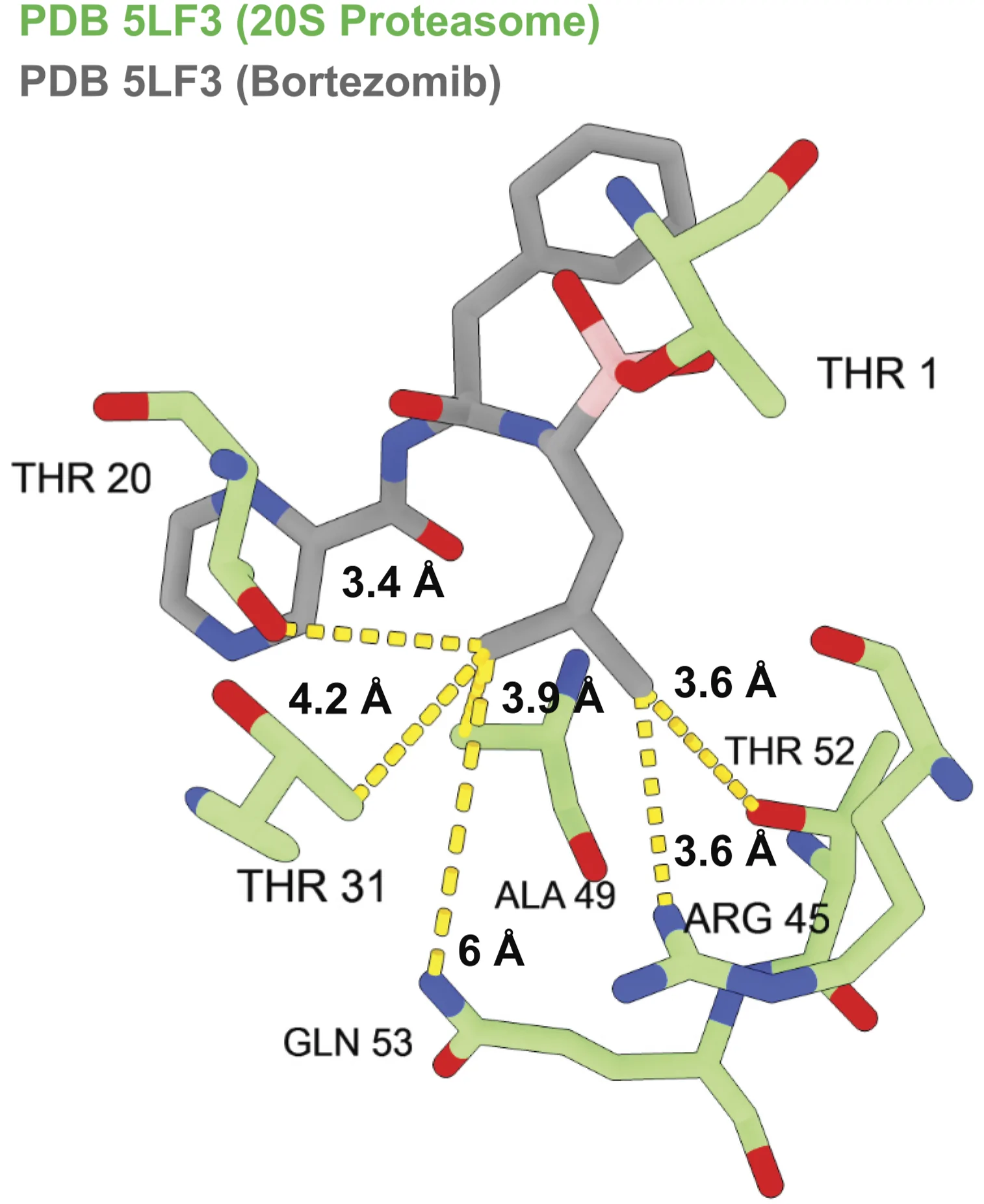
Steric constraints at the bortezomib-binding site in the 20S proteasome. Structural visualization of the bortezomib-bound human 20S proteasome (PDB 5LF3) highlights the spatial arrangement of residues surrounding the isobutyl-binding pocket. Key residues within 3–6 Å of the inhibitor include Thr1, Thr20, Thr31, Ala49, Arg45, Thr52, and Gln53, which form van der Waals contacts (yellow dashed lines). The restricted space in this hydrophobic pocket may disfavor accommodation of bulkier groups such as the phenyl moiety in PZL-26, contributing to the compound’s selectivity for LONP1 over the proteasome.

PZL-26 exhibits a π-stacking interaction between its pyrazine ring and Trp770, alongside two hydrogen bonds with the NH and CO groups of Trp770’s backbone (Fig 2E, with distances highlighted in yellow). The phenyl group occupies a hydrophobic pocket, fitting better than bortezomib’s isobutyl group and similar to compound 12d, thereby generating stronger stabilizing hydrophobic interactions. This feature may contribute to the selectivity of PZL-26 for LONP1, as the 20S proteasome appears to have limited space around the isobutyl-binding site, with surrounding residues located within 3–6 Å (Fig S1), potentially disfavoring the accommodation of a bulkier phenyl group. The morpholine ring is solvent-exposed, facing an area with no direct interactions but surrounded by hydrophilic residues.

### Effect of LONP1 inhibition in human cells and identification of potential target proteins

**Figure 3.**
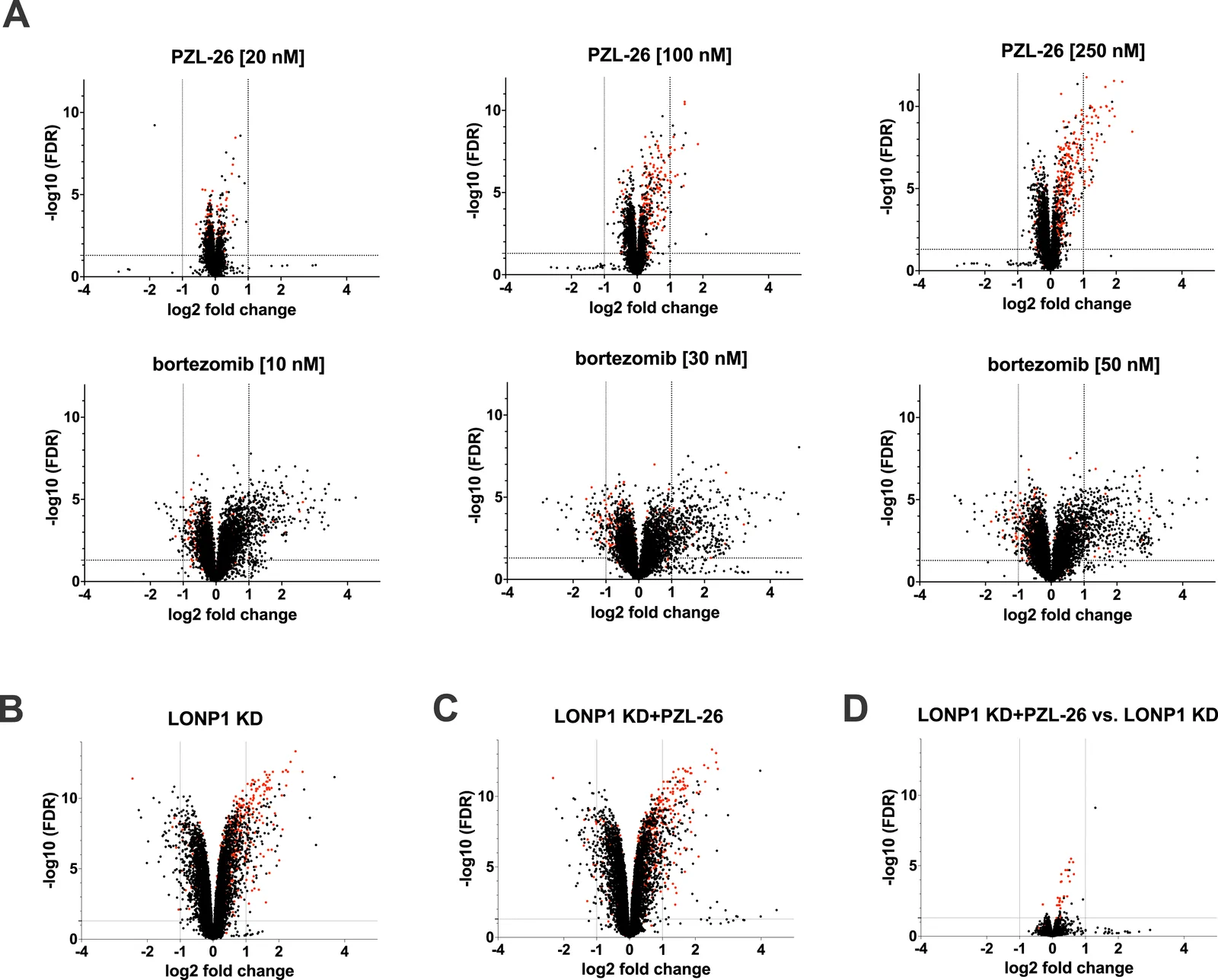
Proteomics analysis after LONP1 knockdown reveals significant overlap with proteins identified after PZL-26 treatment. **(A)** Volcano plots showing the results of quantitative proteomics analysis in 143B human cells after 24-h treatment with PZL-26 (upper panels; 20, 100, and 250 nM) or bortezomib (lower panels; 10, 30, and 50 nM). Mitochondrial proteins are highlighted in red. **(B)** Volcano plot showing protein abundance changes in 143B cells after siRNA-mediated knockdown of LONP1, compared with scrambled siRNA control. Mitochondrial proteins are highlighted in red. **(C)** Volcano plot showing protein abundance changes in 143B cells cotreated with LONP1 siRNA and PZL-26, compared with the scrambled siRNA control. Mitochondrial proteins are highlighted in red. **(D)** Chemical–genetic epistasis: volcano plot comparing LONP1 siRNA + PZL-26 cells with LONP1 siRNA alone. The absence of further accumulation of LONP1-responsive proteins is the predicted signature of an on-target inhibitor. Source data are available for this figure.

**Figure S2.**
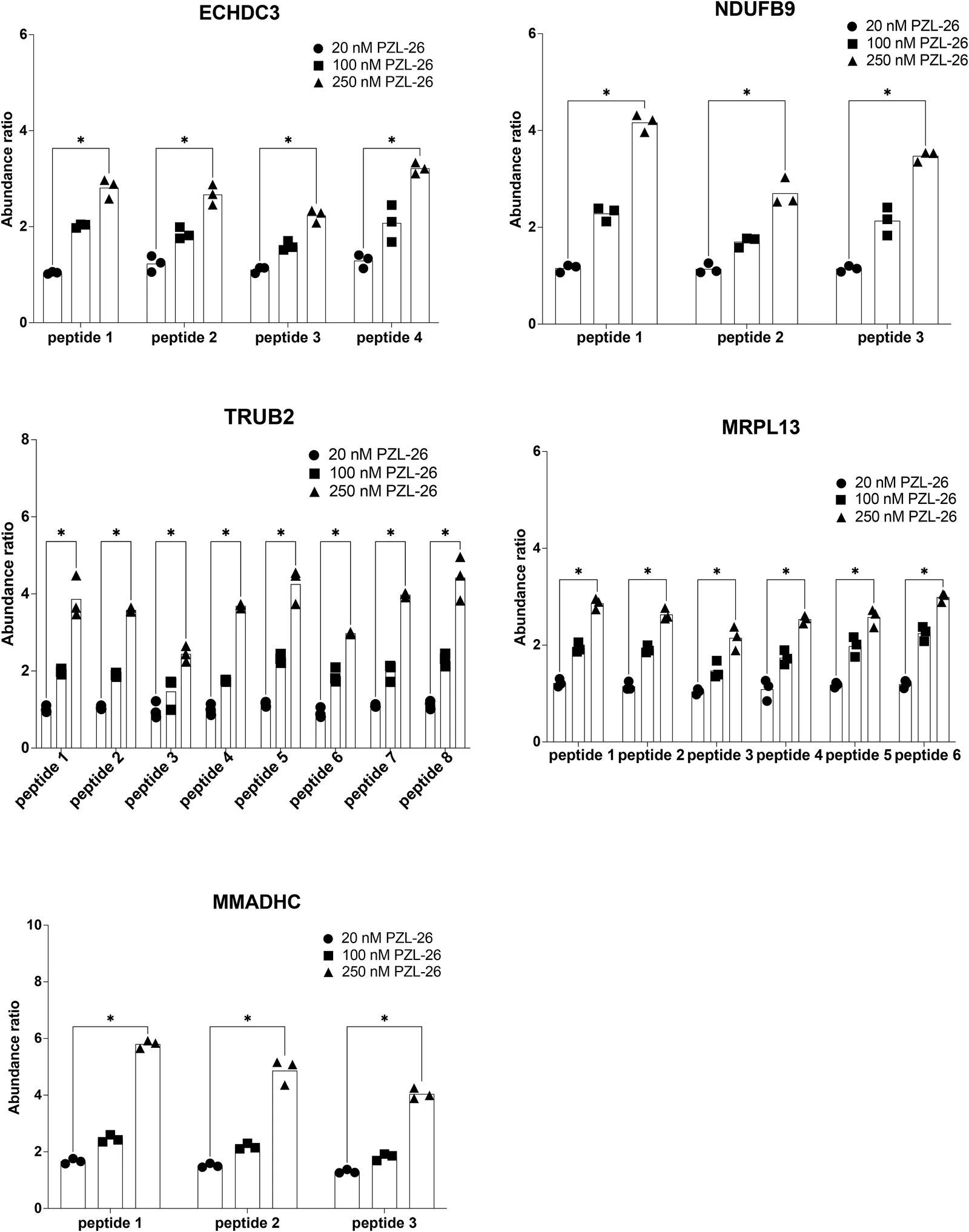
Peptide-level mass spectrometry confirms dose-dependent accumulation of candidate LONP1 substrates. Targeted peptide-level analysis of five candidate LONP1 substrates (ECHDC3, NDUFB9, TRUB2, MRPL13, and MMADHC) within the TMT proteomic dataset described in Fig 3A. For each protein, normalized TMT reporter intensities of independent tryptic peptides are plotted across PZL-26 concentrations (20, 100, 250 nM). Concordant, dose-dependent accumulation across multiple peptides per protein provides peptide-level support for the protein-level quantification. ECHDC3, NDUFB9, TRUB2, and MRPL13 are candidate LONP1 substrates; MMADHC is a direct LONP1 substrate (Fig 4D). Source data are available for this figure.

**Figure S3.**
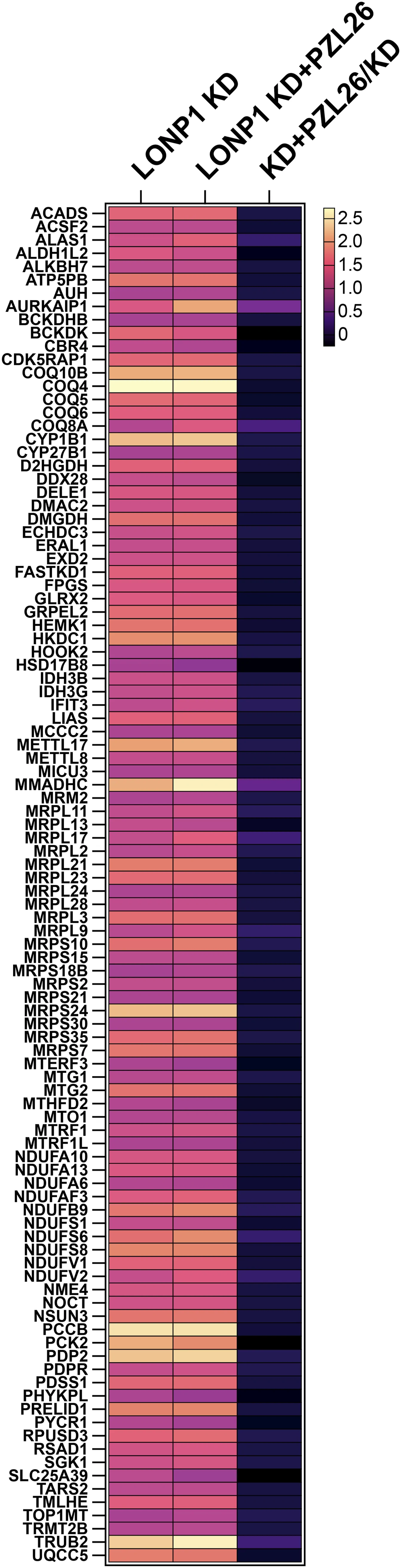
Heatmap of the epistasis experiment confirms that PZL-26 treatment produces no further protein accumulation in LONP1-depleted cells. Heatmap displaying relative protein abundance (log_2_ scale) of proteins up-regulated upon LONP1 knockdown alone (LONP1 KD), combined LONP1 knockdown plus PZL-26 (LONP1 KD + PZL-26), and the corresponding epistasis ratio (KD + PZL-26/KD). Protein intensities are scaled per row. Most of the up-regulated proteins are mitochondrial, including components of OXPHOS (notably complex I) and mitochondrial gene expression pathways. The near-uniform low intensity of the third column reflects the absence of further accumulation upon PZL-26 treatment in LONP1-depleted cells, the predicted signature of on-target inhibition. Source data are available for this figure.

To validate the selectivity and potency of PZL-26 in a cellular context, we treated human osteosarcoma-derived 143B cells with increasing concentrations of PZL-26 for 24 h and performed tandem mass tag (TMT)–based quantitative proteomics using high-resolution Orbitrap mass spectrometry to assess changes in protein abundance. Our results demonstrated that PZL-26 selectively induces the accumulation of mitochondrial proteins (Fig 3A). This accumulation was dose-dependent, with maximal effects observed at 250 nM. Targeted peptide-level mass spectrometry of four candidate substrates (ECHDC3, NDUFB9, TRUB2, and MRPL13) confirmed concordant, dose-dependent accumulation across multiple independent tryptic peptides per protein (Fig S2), providing peptide-level support for the protein-level quantification. LONP1 knockdown with siRNA produced a similar pattern of mitochondrial protein accumulation (Fig 3B). To establish that the proteomic response to PZL-26 reflects on-target LONP1 inhibition, we performed a chemical–genetic epistasis experiment. Cotreatment with PZL-26 in LONP1-depleted cells (Fig 3C) produced no further accumulation of LONP1-responsive proteins compared with siRNA knockdown alone (Figs 3D and S3), a nonadditivity that is the predicted signature of an on-target inhibitor. In contrast, bortezomib treatment (10–50 nM) resulted in broad protein accumulation, with only 3% being mitochondrial proteins (Fig 3A), consistent with its known activity against the proteasome. We conclude that PZL-26 is a potent and selective inhibitor of human LONP1, whereas bortezomib has broader activity and is not suitable for selective LONP1 studies.

**Figure S4.**
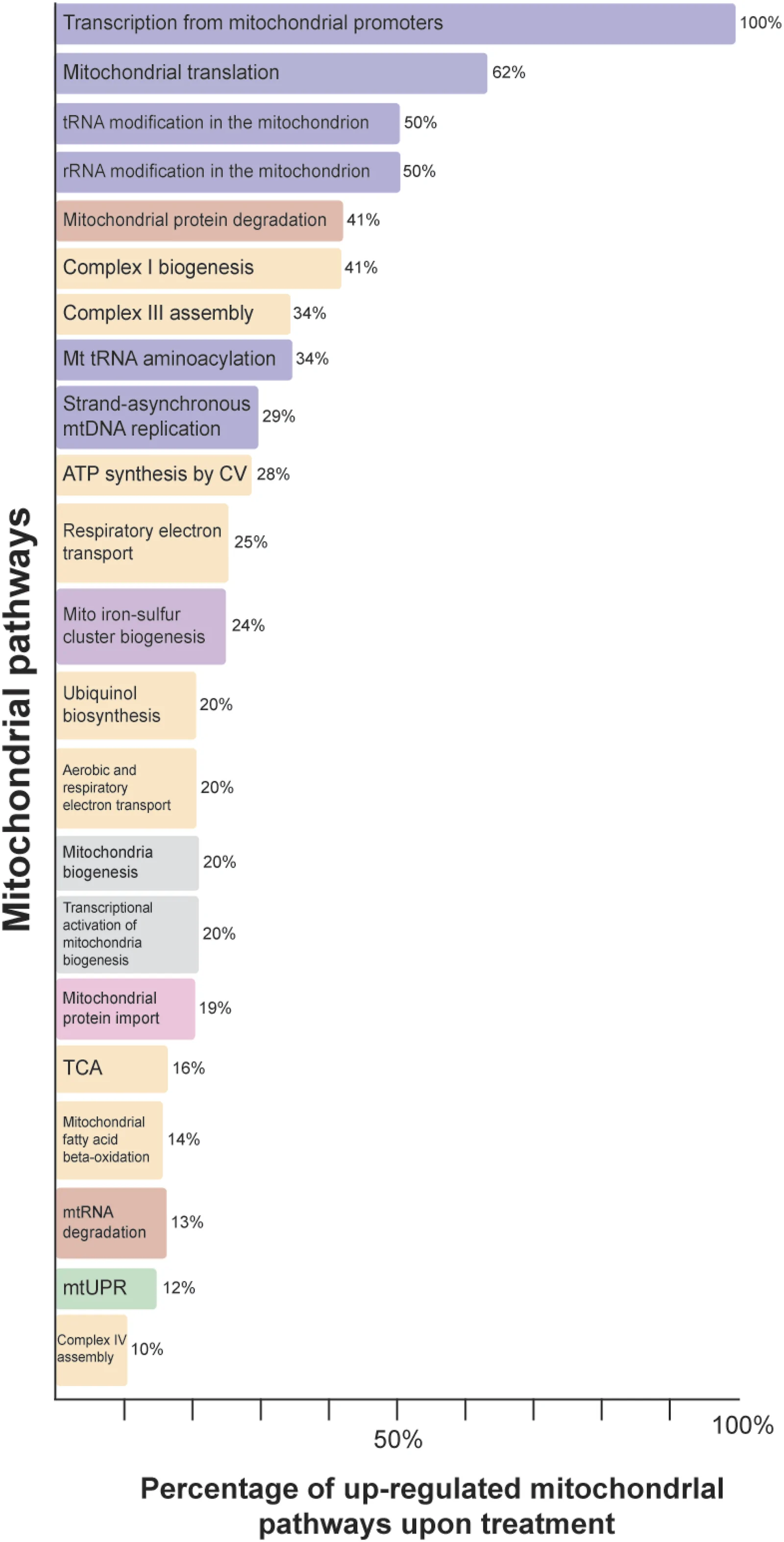
Pathway enrichment analysis of proteins up-regulated upon LONP1 inhibition. Mitochondrial pathways enriched in the proteomic dataset after PZL-26 treatment of 143B cells. Highlighted processes include mitochondrial translation, transcription, protein degradation, and complex I biogenesis, consistent with LONP1’s role in maintaining mitochondrial gene expression and proteostasis. Source data are available for this figure.

We also performed a pathway analysis of the proteins up-regulated in response to PZL-26 treatment in our proteomic dataset (Fig S4). This analysis revealed an accumulation of proteins associated with various mitochondrial functions, including OXPHOS (primarily complex I) and mitochondrial gene expression, encompassing both mitochondrial transcription and translation.

### Cellular target engagement and identification of MMADHC as a LONP1 biomarker

**Figure 4.**
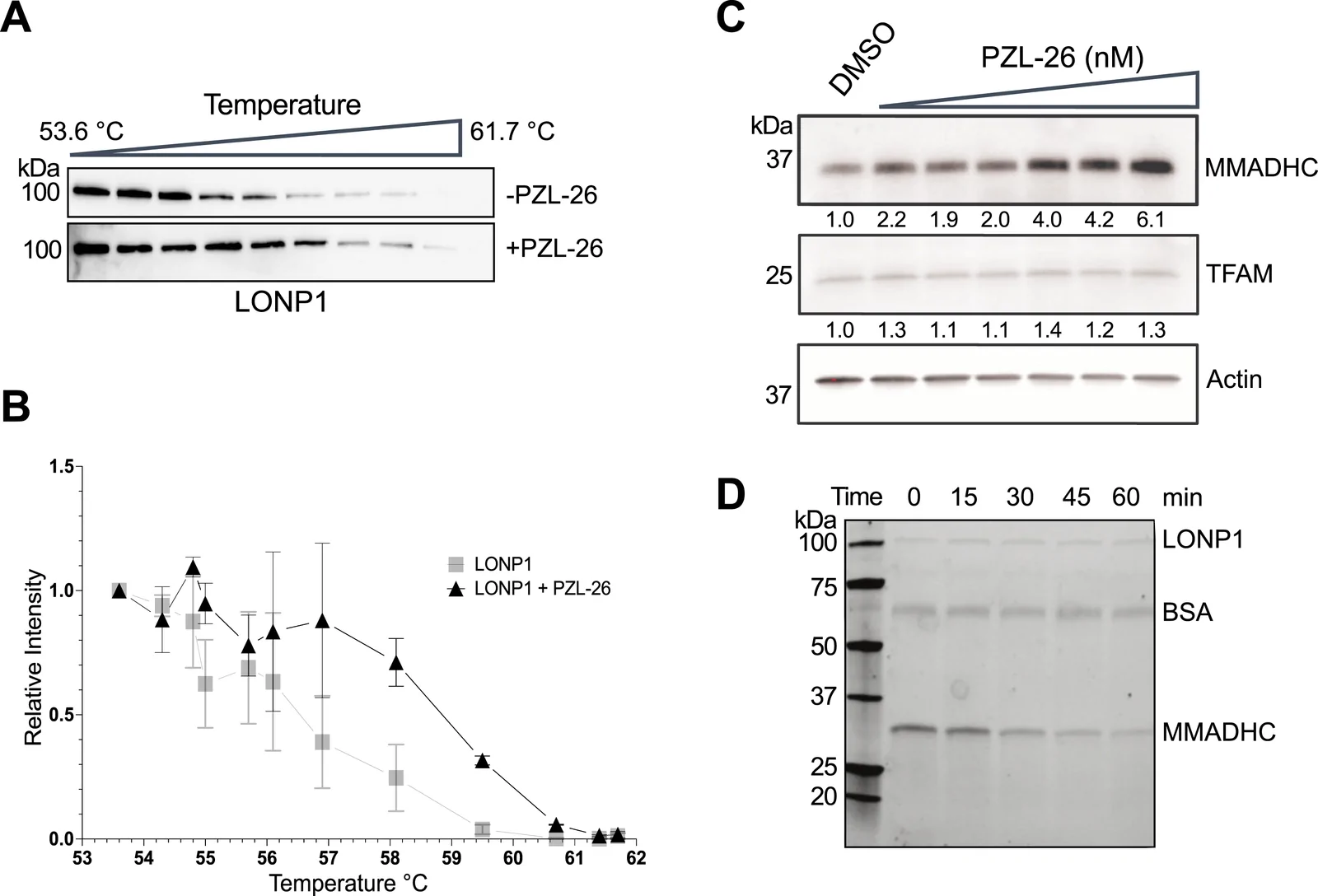
Cellular target engagement by PZL-26 and identification of MMADHC as a LONP1 substrate. **(A)** Cellular thermal shift assay in 143B cells. Cells were treated with PZL-26 or DMSO and heated across a temperature gradient (53.6–61.7°C) before lysis. LONP1 was detected by immunoblot in the soluble fraction at each temperature. **(B)** Quantification of LONP1 band intensities from (A), plotted as relative intensity versus temperature. PZL-26 treatment produces a right shift in the thermal denaturation curve of LONP1, consistent with direct binding of the compound to LONP1 in intact cells. Data are the mean ± SD. **(C)** Western blot analysis after 24-h treatment with PZL-26 (8–250 nM), showing visual and numeric protein levels of TFAM, MMADHC, and actin (loading control). MMADHC accumulates, but no change in TFAM levels is observed. Signal intensities were quantified, and fold changes relative to the DMSO control (set to 1) were normalized to actin. **(D)** MMADHC is a direct LONP1 substrate. Recombinant MMADHC (500 nM) is degraded by LONP1 (100 nM) in an in vitro degradation assay. Source data are available for this figure.

To complement the chemical–genetic data with direct evidence of target engagement, we tested whether PZL-26 binds LONP1 within intact cells using a cellular thermal shift assay. Treatment of 143B cells with PZL-26 produced a clear right shift in the thermal denaturation curve of LONP1 (Fig 4A and B), indicating direct binding of the compound to LONP1 under physiological conditions.

MMADHC accumulates in a dose-dependent manner in cells treated with PZL-26, increasing approximately sixfold at 250 nM (Fig 4C), and is degraded by purified LONP1 in a time-dependent manner in vitro (Fig 4D). These data support MMADHC as a direct LONP1 substrate and a biomarker of LONP1 inhibition.

### TFAM as a LONP1 substrate

**Figure S5.**
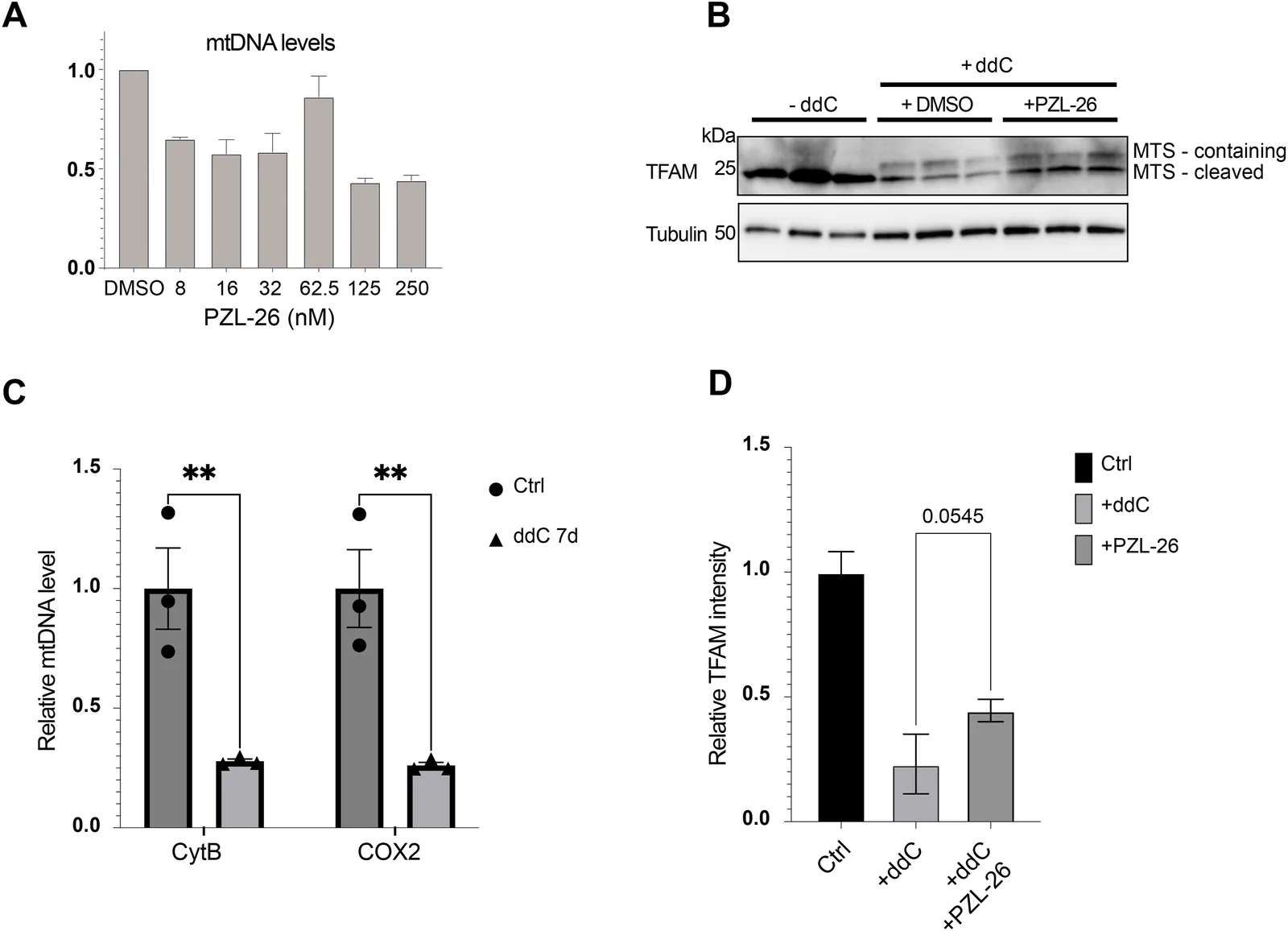
TFAM and mtDNA levels in 143B cells. **(A)** Relative mtDNA levels were quantified by qPCR using primers targeting ND1 and CYTB, normalized to nuclear 18S rRNA, in 143B cells treated with the indicated concentrations of PZL-26. No increase in mtDNA levels was observed (DMSO control normalized to 1.0). n = 2 biological replicates, each with 2 technical replicates; error bars represent the SD. **(B)** TFAM levels in 143B cells after mtDNA depletion by ddC treatment, with or without subsequent PZL-26. Cells were treated with ddH_2_O (Ctrl) or 20 μM ddC for 7 d; on day 7, cells were treated with 0.1% DMSO or 250 nM PZL-26 for 24 h. TFAM levels were assessed by immunoblot, with α-tubulin (Tub1) as loading control. **(C)** Confirmation of mtDNA depletion by ddC. Relative mtDNA levels were quantified by qPCR using primers targeting *CytB* and *COX2*, normalized to nuclear *18S* rRNA, in 143B cells treated with ddH_2_O (Ctrl) or 20 μM ddC for 7 d. ddC reduced mtDNA copy number by ∼70%. **P < 0.01. n = 3 biological replicates, error bars represent the SEM, and significance values were determined by one-way ANOVA relative to Ctrl. **(D)** Quantification of relative TFAM intensity from (B), normalized to Ctrl (Ctrl = 1.0). PZL-26 produced an upward trend in TFAM levels relative to ddC alone (*P* = 0.0545). n = 3 biological replicates, error bars represent the SEM, and significance values were determined by one-way ANOVA relative to Ctrl. Source data are available for this figure.

We next examined TFAM, a well-characterized in vitro substrate of LONP1. However, treatment with PZL-26 did not result in altered TFAM protein levels in 143B cells (Fig 4C). In addition, we observed a slight decrease in mtDNA levels (Fig S5A), likely a downstream consequence of the broader mitochondrial dysfunction caused by LONP1 inhibition, rather than the expected up-regulation that would typically accompany increased TFAM levels. A reduction in mtDNA would be expected to increase the fraction of TFAM that is not bound to DNA and therefore potentially susceptible to LONP1-mediated degradation. When LONP1 is inhibited, stabilization of this DNA-free pool could contribute to the maintenance of total TFAM levels. Previous studies have shown that treatment with bortezomib can lead to stabilization of TFAM in ρ^0^ cells lacking mtDNA (Lu et al, 2013). To test whether TFAM becomes susceptible to stabilization by LONP1 inhibition after mtDNA depletion in 143B cells, we depleted mtDNA by ddC treatment before addition of PZL-26 (Fig S5B). qPCR quantification confirmed that ddC reduced mtDNA copy number by ∼70% in 143B cells (Fig S5C). Under these conditions, PZL-26 produced an upward trend in TFAM levels relative to ddC alone (Fig S5B and D; *P* = 0.0545), consistent with the transient and modest stabilization reported by Lu et al (2013) upon Lon knockdown in mtDNA-deficient cells. Together, these findings are consistent with a model in which LONP1 preferentially degrades DNA-free TFAM.

**Figure S6.**
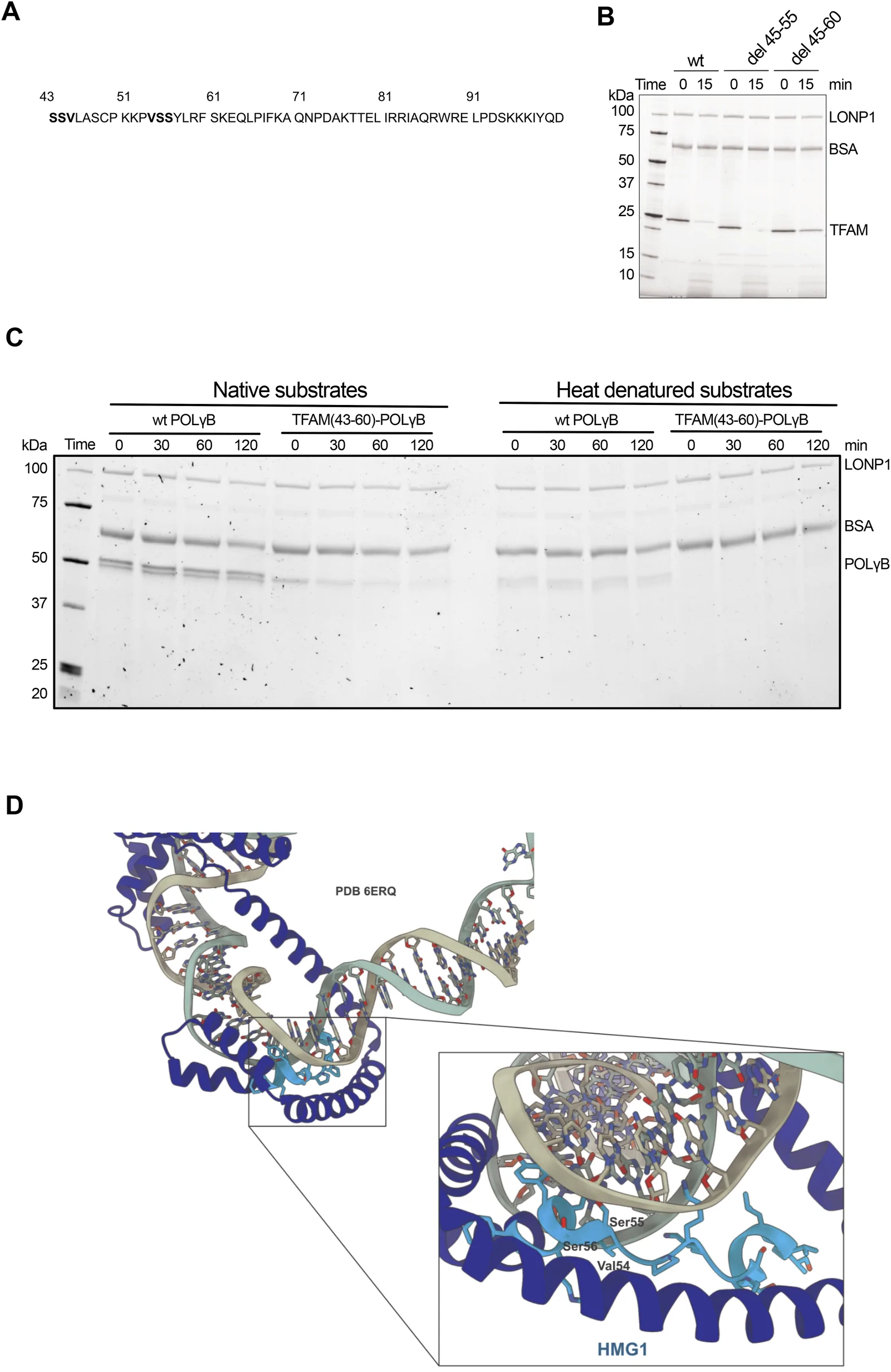
TFAM degron and structural basis for LONP1 targeting. **(A)** Schematic representation of the N-terminal region of mature TFAM (residues 43–100), after cleavage of the MTS. A conserved serine- and valine-rich motif (SSV/VSS) is highlighted in bold. **(B)** Comparison of degradation rates of TFAM variants lacking amino acids 45–55 or 45–60 near the N terminus identifies this region as a critical degron for LONP1-mediated degradation. **(C)** Fusion of the TFAM 43–60 fragment, which includes the serine- and valine-rich motif, to the N terminus of POLγB renders the otherwise resistant protein susceptible to in vitro degradation by LONP1. **(D)** Structure of TFAM bound to DNA, highlighting the HMG1 domain (PDB 6ERQ). The DNA is depicted in light green, whereas the TFAM protein’s secondary structures are shown in blue and cyan. The serine- and valine-rich degron-like sequence (amino acids 43–60), critical for LONP1 degradation, is shown in cyan. Key residues Val54, Ser55, and Ser56 are labeled within the degron-containing region. Ser55 and Ser56 have previously been implicated in phosphorylation-dependent reduction of TFAM-DNA binding (Lu et al, 2013). The boxed inset focuses on this region, illustrating how the folded HMG1 domain (in blue) binds DNA, potentially shielding the degron from LONP1 targeting. This structural arrangement provides a possible explanation for the preferential degradation of DNA-free TFAM, in which the degron-containing region may be more accessible to LONP1. Source data are available for this figure.

We noted that TFAM contains serine- and valine-rich motifs (SSV/VSS) at the mature N terminus of TFAM (amino acids 43–60) (Fig S6A), which resemble motifs involved in substrate degradation by the bacterial Lon protease (Omnus et al, 2023). Indeed, deletion of these motifs blocked TFAM degradation (Fig S6B). Addition of this degron-like sequence also conferred LONP1-mediated degradation to a nonsubstrate protein (in this case, the mitochondrial polymerase gamma B subunit, POLγB), underscoring its functional significance (Fig S6C).

Interestingly, the degron begins in the unstructured N-terminal region of TFAM and continues into the HMG1 domain, which folds upon DNA binding (Fig S6D). Notably, this region includes Ser55 and Ser56, residues within HMG box 1 that have been implicated in phosphorylation-dependent reduction of TFAM-DNA binding and enhanced LONP1-dependent degradation (Lu et al, 2013). This region is involved in DNA interaction (PDB 6ERQ) (Hillen et al, 2017) and is further shielded by a second helix (dark blue), likely obstructing access by LONP1. In the absence of mtDNA, however, this region may remain unstructured, potentially explaining the selectivity of LONP1 for unbound TFAM. Thus, mechanisms that reduce TFAM-DNA association could increase accessibility of the degron-containing region, providing a possible link between regulated changes in TFAM-DNA binding and its susceptibility to LONP1-mediated degradation.

### LONP1 inhibition reveals mitochondrial OXPHOS and proteostasis dependencies

**Figure 5.**
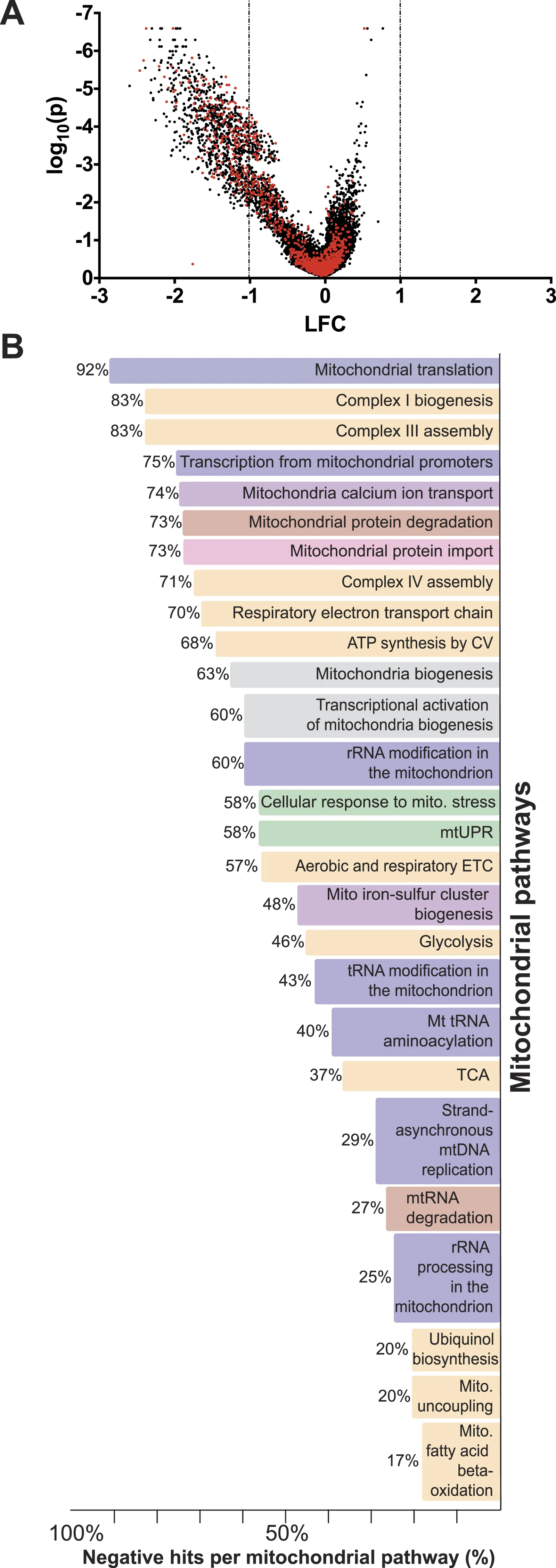
Whole-genome CRISPR-Cas9 genetic screen identifies mitochondrial genes essential for cell survival upon PZL-26 treatment. **(A)** CRISPR guides enriched (positive hits) and depleted (negative hits) in PZL-26–treated RKO cells, compared with an early time point control. Data are plotted as LFC versus log10 of adjusted *P*-value. Genes encoding mitochondrial proteins are highlighted in red. **(B)** Percentage of negative hits categorized by mitochondrial pathways. LFC, log2 fold change. Source data are available for this figure.

**Figure 6.**
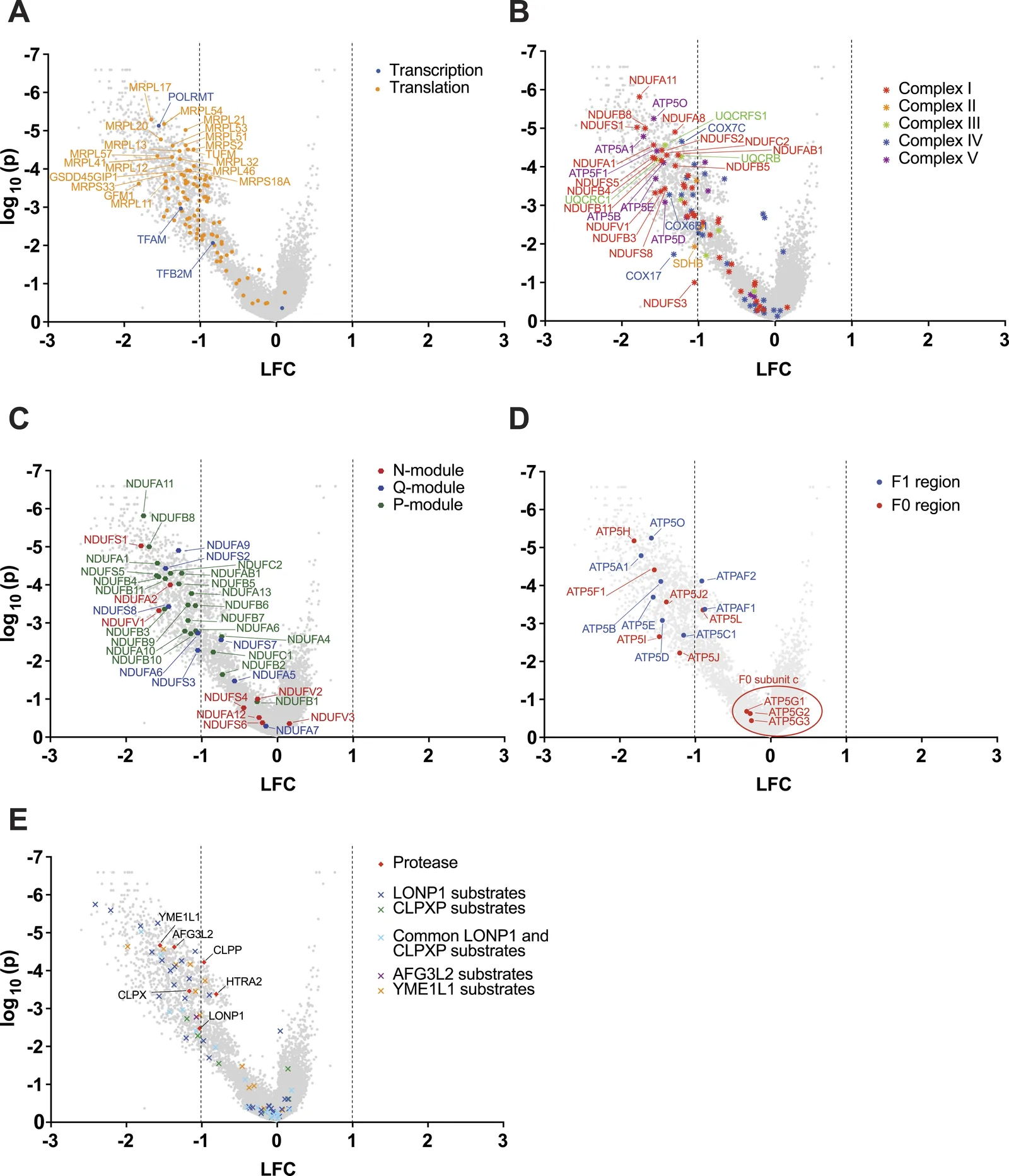
Genome-wide CRISPR screen reveals mitochondrial pathways essential for cell survival under LONP1 inhibition. **(A)** Genes involved in mitochondrial translation (orange) and transcription (blue) whose inactivation decreased cell viability in the presence of PZL-26. **(B)** Genes encoding subunits of OXPHOS complexes: complex I (red), complex II (orange), complex III (green), complex IV (blue), and complex V (purple). **(C)** Complex I subunits grouped by functional modules: N-module (red), Q-module (blue), and P-module (green). Depletion of genes from all modules reduced viability under LONP1 inhibition. **(D)** ATP synthase subunits categorized into F_1_ (blue) and F_0_ (red) regions. Most subunits were essential for cell viability, except for the c-subunit isoforms (ATP5G1–3), which form the membrane-embedded c-ring and did not score as essential. **(E)** Genes encoding mitochondrial proteases (in red) and their known substrates. The screen revealed that inactivation of several proteases, including CLPXP, YME1L1, AFG3L2, and HTRA2, as well as their substrate genes, sensitized cells to LONP1 inhibition. Source data are available for this figure.

To further dissect the cellular dependencies and adaptive responses to PZL-26 treatment, we conducted a whole-genome CRISPR-Cas9 screen in human RKO cells treated with PZL-26. The CRISPR-Cas9 screen enabled us to systematically identify genes whose inactivation negatively impacts cell survival under PZL-26 treatment (Fig 5A and B). The pathway analysis of these genes revealed enrichment in the same mitochondrial processes (Fig 5B) as those observed in our proteomics analysis (Fig S4), that is, mitochondrial translation, transcription, and OXPHOS complexes I-V (Fig 6A–E). This strong overlap between proteomic and genetic screening results underscores the importance of these pathways for cell survival under PZL-26 treatment. Our results suggest that LONP1 inhibition may trigger a compensatory up-regulation of the expression of mitochondrial proteins or lead to the accumulation of defective proteins escaping degradation. Indeed, previous studies have demonstrated that LONP1 loss leads to accumulation of complex I subunits and impaired complex I assembly causing mitochondrial dysfunction (Pryde et al, 2016). In the CRISPR-Cas9 screen, we observed that depletion of complex I subunits belonging to all modules of complex I results in decreased survival of PZL-26–treated cells (Fig 6B and C). Our CRISPR-Cas9 screen showed that nearly all subunits of mitochondrial complex V (ATP synthase) were essential for cell survival after PZL-26 treatment, with the exception of the c-subunit of the Fo region (the c-ring), which did not score as essential (Fig 6D).

Notably, our screen also revealed that depletion of genes for mitochondrial proteases, that is, the ClpXP complex, YME1L1, AFG3L2, and HTRA2, as well as many of their respective substrates (Fig 6E), decreased survival of PZL-26–treated cells. This highlights the crucial role of these mitochondrial proteases and suggests that they have at least partly overlapping functions with LONP1 in maintaining mitochondrial function. In summary, our analysis highlights the extensive impact of LONP1 inhibition on mitochondrial biology, spanning gene expression, OXPHOS, and protein homeostasis.

## Discussion

This study provides novel insights into the role of LONP1 in mitochondrial protein homeostasis and demonstrates the utility of PZL-26 as a selective tool for investigating LONP1 functions. Unlike bortezomib, which inhibits both LONP1 and the proteasome, PZL-26’s selectivity for LONP1 enables more precise investigations into LONP1-specific pathways.

PZL-26 inhibits the proteolytic activity of LONP1, but LONP1 also has other reported activities, including a chaperone function and binding to mtDNA. The chaperone and DNA-binding functions reside in domains distinct from the proteolytic active site and are therefore unlikely to be directly engaged by the inhibitor. Consistent with this, siRNA-mediated knockdown of LONP1, which removes all of its activities, produced a proteomic signature closely resembling that of PZL-26 treatment (Fig 3A and B), and cotreatment of LONP1-depleted cells with PZL-26 gave no further protein accumulation (Fig 3D). The phenotype is thus dominated by loss of proteolytic activity, with no separable contribution from the chaperone or DNA-binding functions detectable at the level of the steady-state proteome, although effects on mtDNA maintenance or nucleoid organization would not necessarily be captured by proteomics and cannot be formally excluded. The cellular phenotypes described here should accordingly be interpreted as consequences of proteolytic inhibition.

Our findings underscore the critical role of LONP1 in maintaining mitochondrial proteostasis and support its involvement in OXPHOS. We observed strong alignment between our CRISPR-Cas9 screen and proteomics data, with both approaches converging on the same mitochondrial pathways. This highlights LONP1’s involvement in both complex I biogenesis and mitochondrial gene expression and emphasizes the consistency between the genetic and proteomic effects of LONP1 inhibition.

One notable observation from the CRISPR screen was that nearly all subunits of complex V (ATP synthase) were required for cell survival under LONP1 inhibition, with the exception of the c-subunit of the Fo region (the c-ring). A possible explanation for this finding is that the c-subunit has a particularly long half-life relative to other mitochondrial proteins, including other subunits of ATP synthase. As a highly stable, membrane-embedded component, the c-ring may rely less on active turnover and LONP1-mediated degradation for its maintenance (Bogenhagen & Haley, 2020). Its stability may render it less susceptible to proteotoxic stress or dysfunctional assembly under LONP1 inhibition, making it uniquely dispensable in this context.

Furthermore, the screen revealed the importance of mitochondrial proteases and their substrates in cell survival during LONP1 inhibition, emphasizing the critical role of mitochondrial proteostasis in cell viability. In particular, we observed functional interactions between LONP1 and other mitochondrial proteases, such as ClpXP (Szczepanowska & Trifunovic, 2022) and AFG3L2 (Banfi et al, 1999; Patron et al, 2018), suggesting compensatory or redundant mechanisms that may help maintain mitochondrial proteostasis when LONP1 is inhibited. This interrelationship suggests that inhibiting multiple proteases simultaneously could have synergistic effects, particularly in cells with high oxidative stress or altered mitochondrial function, such as cancer cells.

Our observations align with recent work by Maneix and colleagues, which demonstrated LONP1’s significant role in conferring resistance to proteasome inhibitors in multiple myeloma (Maneix et al, 2021). Their findings that LONP1 overexpression reduces the efficacy of these inhibitors suggest that LONP1 can compensate for the loss of proteasome activity. The potential for redundancy among mitochondrial proteases reinforces the concept of targeting both the proteasome and mitochondrial proteases like LONP1 to overcome drug resistance in multiple myeloma and potentially other cancers.

The proteomic dataset reported here identifies proteins that accumulate in cells upon PZL-26 treatment, but proteomics alone cannot distinguish direct LONP1 substrates from proteins that accumulate as an indirect consequence of LONP1 inhibition. The chemical–genetic epistasis experiment (Fig 3D) establishes that the proteome-wide response is mediated through LONP1 rather than through off-target proteases, but does not address which individual accumulating proteins are direct substrates. MMADHC is supported as a direct LONP1 substrate by the combination of dose-dependent accumulation in cells (Fig 4C) and time-dependent degradation by purified LONP1 in vitro (Fig 4D). The four additional proteins examined in detail (ECHDC3, NDUFB9, TRUB2, and MRPL13) are described here as candidate substrates pending direct in vitro validation.

In conclusion, our findings validate the selectivity of PZL-26 as a tool for investigating LONP1 and underscore the critical role of LONP1 in mitochondrial proteostasis and drug resistance. The synergistic potential of targeting LONP1 along with other mitochondrial proteases, especially in cancer cells, provides a promising direction for future therapeutic strategies. The insights gained from this study, combined with recent evidence from the field, offer a foundation for further research into LONP1’s broader biological functions and therapeutic potential, particularly in overcoming drug resistance through combined protease inhibition.

## Materials and Methods

### Generation of constructs and protein purification

Deletion mutants of TFAM were generated using a previously published protocol (Hansson et al, 2008), and point mutations were introduced via site-directed mutagenesis (QuikChange Lightning; Agilent) on the wild-type TFAM construct. Recombinant LONP1 (amino acids 68-959) lacking the predicted mitochondrial targeting sequence (MTS; amino acids 1-67) and TFAM (amino acids 43-246) lacking the predicted MTS (amino acids 1-42) were purified as previously described (Gesé et al, 2021 *Preprint*; Mehmedovic et al, 2022). Wild-type POLγB, lacking the predicted MTS (amino acids 1–25), and a variant fused to the TFAM degron-like sequence (TFAM residues 43–60) were expressed and purified as previously described (Fuste et al, 2010). The MMADHC protein (amino acids 12–296), lacking the predicted MTS (amino acids 1–11), was cloned into the pET28-MBP-TEV vector and overexpressed in *Escherichia coli* (Rosetta pLysS) as a 6x-His-MBP-MMADHC fusion protein.

Protein purification followed these steps: Cells were lysed in a buffer containing 20 mM Tris–HCl (pH 8.0), 500 mM NaCl, 10 mM β-mercaptoethanol, and 1% Triton X-100. A maltose-binding protein (MBP) fusion of MMADHC was first purified using Ni^2+^-affinity chromatography, followed by purification with HiTrap Q Sepharose chromatography. TEV protease cleavage was then performed to remove the MBP tag. Subsequently, TEV and MBP were separated from MMADHC using a HiTrap Ni^2+^ column, and the MMADHC protein was further purified using a HiTrap Heparin column.

### Biochemical assays

LONP1 activity and the inhibitory effect of compounds were assessed in degradation assays using either a short peptide or recombinant TFAM protein as substrates. We used the previously described FRET substrate FRETN-89-98Abu (Lee & Berdis, 2001; Fishovitz et al, 2011), consisting of the peptide YRGIT(2Abu)SGRQK, end-labeled with the Dabcyl/5-FAM quencher/fluorophore pair. Cleavage of this peptide results in an increase in fluorescence intensity (excitation 485 nm/emission 520 nm), which is proportional to the activity of LONP1. The assay was developed using a high-throughput 384-well plate platform and performed as follows: assay buffer (10 μl) containing 25 mM Tris–HCl, pH 8.0, 10 mM MgCl_2_, 0.03 mg/ml BSA, 0.5 mM DTT, 0.003% Tween-20, 10 mM NaCl, 60 μM ATP, and 0.5 mM EGTA, and either LONP1 (50 nM final concentration as a hexamer) or buffer (no-enzyme control) was incubated with compounds or 0.1% DMSO for 15 min. Then, 5 μl of FRET peptide substrate (3 μM final concentration) per reaction was added, making the final reaction volume 15 μl. The reaction was incubated for 1 h at 37°C, and fluorescence intensity was measured using PHERAstar Microplate Reader (BMG Labtech). The response was calculated as a ratio between the intensities of the sample (S) and the negative control (NC) or positive control (PC) reaction, using the formula:

$$response=\frac{S-NC}{PC-NC}.$$

The data were plotted and analyzed using GraphPad 10, and IC_50_ values were calculated.

When using recombinant TFAM as a substrate, the enzyme assay was performed similarly to the peptide assay, with the following modifications: the assay buffer contained 2 mM ATP; LONP1 was used at a final concentration of 75 nM (calculated as a hexamer) or replaced with buffer as a no-enzyme control; and TFAM was added at a final concentration of 2 μM. To assess compound effects, 1 μl of compound or DMSO (0.1% final concentration) was added to the reaction (final volume 16 μl). Results were assessed after SDS–PAGE separation and visualization of TFAM protein band intensities.

To test whether MMADHC is a substrate of LONP1, the degradation assay was performed similarly to the TFAM assay, but with 100 nM LONP1 (calculated as a hexamer) and 500 nM MMADHC. The reaction was followed for 1 h, with samples taken every 15 min. The samples were analyzed on SDS–PAGE to separate different protein bands, and MMADHC band intensities were assessed.

### Cell culture

143B osteosarcoma cells (ATCC CRL-8303) were cultured in DMEM low glucose (1 g/liter) medium supplemented with 10% fetal bovine serum (FBS). For all experiments, early passage cells were seeded into 6-well or 12-well plates at a concentration of 100,000 cells/ml or 50,000 cells/ml, respectively. The next day, cells were either treated with compound PZL-26 (synthesized at TCG Lifesciences, Hyderabad, India, under the direction of Pretzel Therapeutics) or bortezomib (504314; Sigma-Aldrich) at the indicated concentrations or DMSO (0.1% final concentration) for 24 h, or transfected with scramble siRNA (D-001810-10-20; Dharmacon) or LONP1-specific siRNA (L-003979-00-005l; Dharmacon) using RNAiMAX reagent (13778150; Thermo Fisher Scientific) according to the manufacturer’s protocol. After 24 h of compound treatment, cells were washed with EBSS buffer and either collected by trypsinization or lysed directly for proteomics analysis. For the chemical–genetic epistasis experiment, after the 48-h transfection with scramble or siLONP1 siRNA, cells were treated for 24 h with 250 nM PZL-26 or DMSO, washed with EBSS buffer, and lysed directly for proteomics analysis. For the CRISPR-Cas9 screen, RKO cells were cultured in DMEM + GlutaMAX (4.5 g/liter D-glucose) supplemented with 10% FBS. For the ddC depletion experiment, 143B cells were seeded in 100-mm cell culture plates and treated with either ddH2O or 20 μM ddC for 4 d. On day 4, the cells were trypsinized, quantified, and seeded in 6-well plates at 0.5 × 10^5^ cells/ml and fresh ddC was added to continue treatment for 3 more days. On day 7, all media were replaced with fresh 10% FBS/DMEM with either 0.1% DMSO (Ctrl) or 250 nM PZL-26. After 24 h, the cells were collected and pelleted. 1/5 of each sample was taken for qPCR analysis of mtDNA levels, and protein isolation and immunoblotting were performed on the remaining cell pellet, as subsequently described.

### CRISPR-Cas9 screening

The human colon carcinoma cell line RKO (ATCC CRL-2577) was transduced to express the Cas9 nuclease as detailed in Schmierer et al (2017). Subsequently, the cells were transduced with the genome-wide Brunello sgRNA library (Addgene), containing 77,441 sgRNAs targeting nearly every human gene with four sgRNAs per gene, together with 1,000 nontargeting control sgRNAs (Doench et al, 2016), at an MOI of 0.3 (>500 cells/guide) in 4 μg/ml of polybrene. Once transduced and selected with puromycin (2 μg/ml) for 2–7 d, the cells were allowed to grow without selection. The day before the start of the treatment, 1.2 × 10^8^ cells were seeded. The next days, the medium was removed and replaced with fresh medium containing either DMSO or 500 nM PZL-26. After 7 d of treatment, viable cells were harvested and sent to the CRISPR Functional Genomics facility in Karolinska Institutet where DNA was isolated and guide sequences were amplified by PCR. Analysis was performed using the Reactome database (Milacic et al, 2024).

### Protein extraction and Western analysis

Cells collected for Western analysis were lysed using RIPA lysis buffer (Thermo Fisher Scientific) supplemented with protease inhibitors (1 mM phenylmethylsulfonyl fluoride, 2 mM pepstatin A, 0.6 mM leupeptin, and 2 mM benzamidine). Samples were rotated at 4°C for 30 min to facilitate lysis and then centrifuged at 13,523*g* for 3 min. The supernatant was collected, and protein concentrations were determined using Pierce BCA Protein Assay (Thermo Fisher Scientific). 10–20 μg of protein was separated by SDS–PAGE, transferred to nitrocellulose membranes (Amersham), and immunoblotted with antibodies against TFAM (Shi et al, 2012), MMADHC (23191-1-AP; Proteintech), β-actin (ab6276; Abcam), α-tubulin (T5168; Sigma-Aldrich), and LONP1 (ab224316; Abcam). Unless otherwise indicated, all primary antibodies were used at a dilution of 1:1,000. Anti-α-tubulin and anti-β-actin antibodies were used at a dilution of 1:5,000. Bands were imaged and analyzed using Bio-Rad ChemiDoc MP Imaging System.

### Cellular thermal shift assay

143B cells were seeded at 3 × 10^6^ cells per T-175 flask and allowed to attach overnight. The next day, cells were treated with 250 nM PZL-26 or 0.1% DMSO (vehicle control) and incubated for 24 h. Cells were harvested by trypsinization, counted, and pelleted by centrifugation at 1,000*g* for 5 min. Pellets were washed twice with PBS and resuspended in PBS supplemented with protease inhibitors to ∼1 × 10^6^ cells per 60 μl. Aliquots of 60 μl were distributed into 16 PCR strip tubes per condition and heated across a temperature gradient spanning 53.6–61.7°C for 3 min in a qPCR thermocycler. Samples were immediately placed on ice, 30 μl PBS supplemented with protease inhibitors was added, and cells were subjected to two freeze–thaw cycles in liquid nitrogen. Samples were transferred to Eppendorf tubes and centrifuged at 20,000*g* for 20 min at 4°C. The soluble supernatant (60 μl) was collected and analyzed by immunoblotting for LONP1, as described above.

### Relative quantification proteomics

The samples were homogenized in lysis buffer containing 2% sodium dodecyl sulfate by ultrasonication for 30 min. The protein concentration in the supernatants was determined using Pierce BCA Protein Assay (Thermo Fisher Scientific). Aliquots containing 20–50 μg of each sample were reduced using DTT and alkylated (either methyl methanethiosulfonate or iodoacetamide). Proteins were digested by two sequential additions of trypsin. Peptides were collected and labeled using TMTpro isobaric mass tagging reagents (Thermo Fisher Scientific) according to the manufacturer’s instructions. The labeled samples of one TMT set were pooled in equal amounts, and peptides were purified using HiPPR Detergent Removal Kit and Pierce Peptide Desalting Spin columns (both from Thermo Fisher Scientific). The combined labeled TMT sets were fractionated via basic reversed-phase chromatography on the Dionex Ultimate 3000 UPLC system (Thermo Fisher Scientific) on a Waters XBridge BEH C18 column. Peptides were eluted during a nonlinear gradient with increasing acetonitrile concentration from 3 to 50%. The initial fractions were concatenated into 22–36 final fractions, dried by SpeedVac, and reconstituted in 3% acetonitrile, 0.2% formic acid for analysis.

The fractions were analyzed on an Orbitrap Tribrid mass spectrometer equipped with the FAIMS Pro ion mobility system and interfaced with a liquid chromatography system (Thermo Fisher Scientific). Peptides were separated on an in-house packed analytical column (40 cm × 75 μm, particle size 3 μm, ReproSil-Pur C18, Dr. Maisch) using a stepped gradient from 4% to 80% acetonitrile in 0.2% formic acid over 80 min at a flow rate of 300 nl/min. FAIMS Pro was alternating between compensation voltages of −50 and −70, and the same data-dependent settings were used at both compensation voltages. The precursor ion mass spectra were acquired at a resolution of 120,000 and an m/z range of 375–1,500. Using a cycle time of 1.5 s, the most abundant precursors with charges 2–7 were isolated with an m/z window of 0.7 and fragmented by collision-induced dissociation at 35%. Fragment spectra were recorded in the ion trap at a rapid scan rate. The 10 most abundant MS2 fragment ions were isolated using multinotch isolation for further MS3 fragmentation. MS3 fragmentation was performed using higher energy collisional dissociation at 55%, and the MS3 spectra were recorded in the Orbitrap at 50,000 resolution and an m/z range of 100–500.

Identification and relative quantification were performed using Proteome Discoverer version 2.4 (Thermo Fisher Scientific). Database matching was performed against the *Homo sapiens* Swiss-Prot database with a precursor tolerance of 5 ppm and a fragment ion tolerance of 0.6 Da. Tryptic peptides were accepted with 1 missed cleavage; methionine oxidation was set as a variable modification, and cysteine alkylation, TMTpro on lysine, and peptide N termini were set as fixed modifications. Percolator was used for PSM validation with a strict FDR threshold of 1%. For quantification, TMT reporter ions were identified in the MS3 higher energy collisional dissociation spectra with a 3 mmu mass tolerance, and the TMT reporter intensity values for each sample were normalized to the total peptide amount. SPS Mass Match threshold was set to 65%. Only unique peptides were used for relative quantification.

Peptide-level quantification was performed on the same dataset to provide peptide-level support for selected candidate substrates (ECHDC3, NDUFB9, TRUB2, MRPL13). For each protein, normalized TMT reporter intensities of all uniquely mapped tryptic peptides were extracted from Proteome Discoverer and plotted across PZL-26 concentrations to assess concordance of dose-dependent accumulation across independent peptides (Fig S2).

### mtDNA-level determination by qPCR

Total DNA was isolated from 5 to 10 × 10^5 143B cells treated with varying concentrations of PZL-26 or DMSO (control) using High Pure PCR Template Preparation Kit (Roche), or by suspending the cells in 250 μl of lysis buffer (25 mM Hepes (pH 7.6), 150 mM NaCl, 1 mM EDTA (pH 8.0), 0.5% (vol/vol) Triton X-100, and 0.1 mg/ml Proteinase K), incubated at 55°C for 1 h, and genomic DNA was extracted using a Zymo Genomic DNA Clean and Concentrator (D4010; Zymo Research), following the manufacturer’s protocol. The DNA was used as a template in qPCRs targeting the mtDNA genes *ND1*, *CYTB*, and *COX2*, with the nuclear 18S rDNA gene used as a control. Primer sequences were as follows: ND1 forward, 5′-CCC TAA AAC CCG CCA CAT CT-3′; ND1 reverse, 5′-GAG CGA TGG TGA GAG CTA AGG T-3′; CYTB forward, 5′-ACA ATT CTC CGA TCC GTC CC-3′; CYTB reverse, 5′-GTG ATT GGC TTA GTG GGC GA-3′; COX2 forward, 5′- CAG ACG AGG TCA ACG ATC CC-3′; COX2 reverse, 5′- TCA ACG TCA AGG AGT CGC AG -3′; 18S rDNA forward, 5′-AGA AAC GGC TAC CAC ATC CA-3′; and 18S rDNA reverse, 5′-CCC TCC AAT GGA TCC TCG TT-3′. Relative quantification of the Ct values was done compared with the nuclear control, and the values were also normalized to DMSO-treated samples (value 1). The normalized ND1 and CYTB values were averaged and are shown in Fig S5A along with the SD. The experiment was performed with two biological replicates and two technical replicates for each sample.

### LONP1 proteolysis assay

The protease activity of purified LONP1 on TFAM variants was measured in 15 μl reaction volumes containing 55 nM LONP1 (calculated as a hexamer) and 500 nM of the respective TFAM variants. Wild-type POLγB and a POLγB variant containing a TFAM-like degron sequence were also analyzed under the same conditions, using 500 nM protein in each reaction. Samples were incubated at 37°C for 0–60 min in a buffer containing 50 mM Tris–HCl (pH 8.0), 10 mM MgCl_2_, 0.1 mg/ml BSA, 2 mM ATP, and 1 mM DTT. Reactions were stopped by the addition of Laemmli sample buffer (Bio-Rad). The samples were then run on precast 4–20% gradient SDS–PAGE gels (567–8094; Bio-Rad) and visualized using ImageLab software (Bio-Rad) to assess proteolytic activity on TFAM. Band intensities were measured with ImageLab, and calculations were performed to determine the percentage of remaining TFAM. All reactions and calculations were performed in triplicate, and SDs were calculated.

### Cryo-EM sample preparation and data acquisition

Purified LONP1 (7.5 μM calculated as a monomer) was thawed and dialyzed into a buffer containing 50 mM NaCl, 25 mM Tris, pH 8.0, 1% glycerol, and 1 mM DTT in 200 ml for 2 h. A sample mix (10 μl) containing the dialyzed protein (5 μM), 10 mM MgCl_2_, 2 mM ATP, and PZL-26 to a final inhibitor:protein monomer ratio of 20:1 was incubated at RT for 15 min. A 3.5 μl aliquot of the sample mix was applied to glow-discharged UltrAuFoil R2/2 200 mesh Au grids (Quantifoil Micro Tools) and vitrified in liquid ethane (Vitrobot Mark IV System, Thermo Fisher Scientific). Grids were clipped afterward and stored in liquid nitrogen until they were screened.

### Cryo-EM data acquisition

The selected grid was transferred under cryogenic conditions into the TFS Titan Krios TEM with an acceleration voltage of 300 kV. Micrographs were acquired on the Gatan K3 camera under low-dose conditions with a total electron dose of 40.4 e−/Å^2^. Data were collected with underfocus values ranging from 0.4 to 2.8 μm at a nominal magnification of 105,000×, resulting in a pixel size of 0.833 Å/px. Automated data acquisition was done by SerialEM in fringe-free imaging mode using 3 × 3 beam shift pattern and 5 exposures per hole in a movie mode, and 4-s exposure was recorded into 40 individual frames. The forty-frame movies were then globally and locally (6 × 6 patches, 20% patch overlap) aligned to compensate for drift and beam-induced motion using the program motioncor2. The resulting dose-weighted sum of aligned frames was used for subsequent image processing steps, except for estimating the contrast transfer function parameters, which were estimated from aligned, non–dose-weighted micrographs using the program gCTF.

### Asymmetric reconstruction of the LONP1 protein

Further data processing was done using internally modified software stack based on the SCIPION 3.0.9 package. Two sets of dose-weighted LONP1 micrographs (4154 and 2725) were processed separately until the 3D initial model generation step. For initial processing, the micrographs were 4x downsampled. The SPHIRE-crYOLO protocol was used for particle picking of 2,661,462 and 248,610 particles (80 × 80 pixel box) from each set of “denoised” micrographs with artificially increased signal-to-noise ratio by the JANNI protocol. The particles were subjected to the three rounds of RELION 2D classification, and only particles from high-quality 2D class averages were selected. Those sets of particles were run through xmipp3-remove duplicates protocols and particles from the two acquisitions were joined together. Those joined sets resulting in 217,111 centered particles were re-extracted from the micrographs and run through the final set of RELION 2D classification resulting in 209,371 clean particles. The initial 3D model was de novo–generated by the RELION 3D initial model algorithm, which was used as an input to a set of unmasked RELION 3D-autorefine runs without imposed symmetry. Centered LONP1 particles were re-extracted from unbinned micrographs (320 × 320 pixels) and subjected to further data processing. Multiple rounds of the RELION contrast transfer function refinement and Bayesian polishing together with masked RELION 3D-autorefine were applied. The final RELION “3D-autorefine” was followed by the RELION “post-processing” protocol generating final 3D volume for model building. The post-processing used MTF correction but no B-factor sharpening.

## Supplementary Material

Reviewer comments

## Data Availability

The atomic models and cryo-EM density maps have been deposited in the Protein Data Bank and the Electron Microscopy Data Bank under the following accession codes: human wild-type LONP1 bound to PZL-26, PDB 31SY and EMDB EMD-58651. The mass spectrometry and Proteome Discoverer analysis files have been deposited in the MassIVE repository under dataset identifier MSV000102843. The CRISPR-Cas9 screening data have been deposited in the Gene Expression Omnibus under the accession number GSE343387.
