# Supplementary material for: A selective inhibitor reveals roles for LONP1 in mitochondrial proteostasis and OXPHOS dependencies
Source: Life Sci Alliance. 2026 Oct 1;9(12):e202503535. doi: 10.26508/lsa.202503535 (PMC13631567; doi:10.26508/lsa.202503535)
Supplement: Supplementary file 1 [file LSA-2025-03535_review_history.pdf]

A Selective Inhibitor Reveals Roles for LONP1 in Mitochondrial Proteostasis and Cellular Metabolism

Zsolt Szilagyi, Pauline Michon, Carlos Pardo-Hernández, Madalina Sacultanu, Javier Miralles Fusté, Andrew Griffin, Paul Charifson, Cindy Phan, Yonghong Shi, Gunther Kern, Gabriel Martinez Botella, Thomas Keating, Bertil Macao, Nils-Göran Larsson, Maria Falkenberg, Jeremy Green, and Claes Gustafsson
DOI: https://doi.org/10.26508/lsa.202503535

Corresponding author(s): Claes Gustafsson, University of Gothenburg; Maria Falkenberg, University of Gothenburg; and Jeremy Green,

Review Timeline:

Submission Date:	2025-10-15
Editorial Decision:	2025-12-24
Revision Received:	2026-06-22
Editorial Decision:	2026-07-21
Revision Received:	2026-09-02
Accepted:	2026-09-09

Scientific Editor: Sarita Hebbar

Transaction Report:

(Note: With the exception of the correction of typographical or spelling errors that could be a source of ambiguity, letters and reports are not edited. The original formatting of letters and referee reports may not be reflected in this compilation.)

December 24, 2025

Re: Life Science Alliance manuscript #LSA-2025-03535-T

Prof. Claes M Gustafsson
University of Gothenburg
Department of Medical Biochemistry and Cell Biology
P.O. Box 440
Gothenburg 405 30
Sweden

Dear Prof. Gustafsson,

Thank you for submitting your manuscript entitled "A Selective Inhibitor Reveals Roles for LONP1 in Mitochondrial Proteostasis and Cellular Metabolism" to Life Science Alliance. We apologise for the delay in communicating our decision due to editor availability issues.

Your manuscript was reviewed by two expert reviewers whose comments are appended below. As you will note, the reviewers acknowledge the significance of this work. That said, they both have highlighted significant concerns that preclude publication at this stage.

We agree with both reviewers that some validation experiments are required to strengthen the findings presented in this manuscript. In this regard, we draw your attention to Reviewer 1, comment 2 to provide additional western blotting data, or any other relevant data, in support of the proteomics results. Likewise we highlight the comments of Reviewer 2 on the specificity of LONP1-specific inhibitors and agree that you address their concern with any of the suggested approaches.
We also concur with both reviewers that the results must be stated and discussed accurately, and properly contextualised. Further, we request you to carefully re-evaluate the conclusions of presented results and moderate your claims as indicated by Reviewer 2.

In line with their overall assessment, we invite you to submit a revised manuscript addressing the reviewers' comments. When submitting the revision, please include a letter addressing the reviewers' comments point by point. While a rebuttal must respond to all points in some form, additional experiments to resolve these points, other than indicated above, will not be required.

I would be happy to discuss the revision in more detail via email or phone/videoconferencing. Please let me know which option you prefer, if any.

To upload the revised version of your manuscript, please log in to your account: https://lsa.msubmit.net/cgi-bin/main.plex

You will be guided to complete the submission of your revised manuscript and to fill in all necessary information. Please get in touch in case you do not know or remember your login name.

The typical timeframe for revisions is three months. Please note that papers are generally considered through only one revision cycle, so strong support from the referees on the revised version is needed for acceptance.

While you are revising your manuscript, please also attend to the below editorial points to help expedite the publication of your manuscript. Please direct any editorial questions to the journal office.

Thank you for this interesting contribution to Life Science Alliance. We hope that the comments below will prove constructive as your work progresses, and we are looking forward to receiving your revised manuscript.

Sincerely,

Sarita Hebbar, PhD
Scientific Editor
Life Science Alliance
http://www.lsajournal.org

A. THESE ITEMS ARE REQUIRED FOR REVISIONS

-- A letter addressing the reviewers' comments point by point.

-- An editable version of the final text (.DOC or .DOCX) is needed for copyediting (no PDFs).

-- High-resolution figure, supplementary figure and video files uploaded as individual files: See our detailed guidelines for preparing your production-ready images, https://www.life-science-alliance.org/authors

-- Summary blurb (enter in submission system): A short text summarizing in a single sentence the study (max. 200 characters including spaces). This text is used in conjunction with the titles of papers, hence should be informative and complementary to the title and running title. It should describe the context and significance of the findings for a general readership; it should be written in the present tense and refer to the work in the third person. Author names should not be mentioned.

-- By submitting a revision, you attest that you are aware of our payment policies found here: https://www.life-science-alliance.org/copyright-license-fee

B. MANUSCRIPT ORGANIZATION AND FORMATTING:

Full guidelines are available on our Instructions for Authors page, https://www.life-science-alliance.org/authors

We encourage our authors to provide original source data, particularly uncropped/-processed electrophoretic blots and spreadsheets for the main figures of the manuscript. If you would like to add source data, we would welcome one PDF/Excel-file per figure for this information. These files will be linked as supplementary "Source Data" files.

IMPORTANT: It is Life Science Alliance policy that if requested, original data images must be made available. Failure to provide original images upon request will result in unavoidable delays in publication. Please ensure that you have access to all original microscopy and blot data images before submitting your revision.

Reviewer #1 (Comments to the Authors (Required)):

Szilagyi et al., present an interesting study examining the role of LONP1 in mitochondrial protein homeostasis and demonstrating the utility of PZL-26 as a selective tool for investigating LONP1 functions. The study reveals that the interdependence of LONP1 and OXPHOS function in maintaining mitochondrial function by utilizing the CRISPR-Cas9 screen. Furthermore, the authors also found functional interactions between LONP1 and other mitochondrial proteases, such as ClpXP and AFG3L2, highlighting the compensatory mechanisms that may maintain mitochondrial proteostasis and cell survival under LONP1 deficiency. Overall, this study provides novel insights into the specificity of PZL-26 as a tool for investigating LONP1 and highlighting LONP1's critical role in mitochondrial proteostasis and drug resistance. The synergistic potential of targeting LONP1 along with other mitochondrial proteases, especially in cancer cells, provides a promising direction for future therapeutic strategies. I have a few comments:
Comments:
1. Given multiple functions (chaperone activity, protease, and mtDNA maintenance) of LONP1, it would be valuable to discuss which LONP1 activity is inhibited by compound PZL-26.
2. The figure 3 in this manuscript should provide a validation of quantitative proteomics by another biochemical approach, such as Western Blotting.
3. The manuscript should offer a clear explanation why there is no change in the level of TFAM while a reduction in mtDNA levels.

Reviewer #2 (Comments to the Authors (Required)):

The manuscript by Szilagyi et al., "A Selective Inhibitor Reveals Roles for LONP1 in Mitochondrial Proteostasis and Cellular Metabolism," pursues three lines of inquiry: (1) discovery of PZL-26, a selective small-molecule LONP1 inhibitor (primary focus); (2) use of PZL-26 to probe LONP1's role in mitochondrial proteostasis; and (3) assessment of LONP1-dependent turnover of mitochondrial transcription factor A (TFAM). Although the study attempts to integrate these aims, it ultimately does not establish PZL-26 as a specific, selective LONP1 inhibitor in physiologically relevant cell models. Such demonstration would justify using PZL-26 to investigate the impact of LONP1 inhibition as compared to its knockdown on mitochondrial proteostasis, which has been done previously in several studies. The emphasis on TFAM appears tangential, particularly since TFAM is not degraded by LONP1 in the cells systems employed. The work would be strengthened by direct evidence of cellular target engagement/occupancy of mitochondrial LONP1 by PZL-26 and by demonstration of dose- and time-dependent stabilization of validated, LONP1-specific substrates. Below are comments/suggestions for improvement.

Section: "Optimization of LONP1-specific inhibitors".

Cellular target engagement and selectivity needs to be demonstrated. The nanomolar inhibition of purified LONP1 by PZL-26, together with cryo-EM density for PZL-26 in the proteolytic active site, is compelling. The authors are well-positioned to validate PZL-26 as a selective LONP1 inhibitor in cells. Currently, the study lacks direct evidence demonstrating mitochondrial LONP1 engagement under physiological conditions (e.g., in cultured cells). Because off-target effects are common, PZL-26 could also affect other mitochondrial or cellular proteases. Notably, bortezomib has been reported to inhibit LONP1 and additional mitochondrial proteases, insights that informed development of more selective proteasome inhibitors. Cell-based experiments to substantiate on-target action and help to rule out off-target effects could include rescue experiments expressing LONP1 wild-type or variants refractory to PZL-26, or proteomic approaches to identify cellular proteins interacting with PZL-26.

Accurate attribution of the FRET substrate. The Methods state, "A FRET-based assay was developed using the peptide YRGIT(2Abu)SGRQK, end-labeled with the Dabcyl/5-FAM fluorophore/quencher pair," implying novelty. However, this peptide substrate FRETN-89-98Abu was reported previously by Lee and colleagues (Fishovitz et al., 2011; Lee and Berdis, 2001). Please revise the text to credit prior work (e.g., "we used the previously described FRET substrate ..."). This correction is important for proper attribution and for readers to contextualize potency measurements across studies.

Section: "LONP1 inhibition does not affect TFAM protein levels in 143B cells".
MMADHC as a putative LONP1 substrate. the manuscript states: "In agreement with published results, MMADHC ... has significant accumulation in cells treated with X (Fig. 4A)," citing Froese et al., 2015. However, Froese et al. do not address LONP1 or MMADHC proteolysis; thus, the citation does not establish prior precedent for MMADHC as a LONP1 substrate. Please either (1) provide a reference that specifically links MMADHC turnover to LONP1, or (2) revise the text to avoid implying prior evidence.
In vitro data presented showing degradation of recombinant MMADHC by purified LONP1 supports the possibility that MMADHC is a direct substrate. However as written, the claim that these data "reinforce its role as a direct target of LONP1 and a potential biomarker for protease activity" overstates the conclusion. Consider tempering to: "support MMADHC as a candidate LONP1 substrate and a putative biomarker," pending cellular target-engagement evidence.

Section: "Effect of LONP1 inhibition in human cells and identification of potential target proteins".
Limited convergence of PZL-26 inhibition with genetic perturbation. A 55% overlap in protein accumulation profiles between LONP1 siRNA knockdown and PZL-26 treatment Fig 3C) is suggestive but not decisive. Partial overlap could reflect off-target effects, different perturbation magnitude/kinetics, or secondary effects. True on-target inhibitors typically show non-additive/epistasis with the matching genetic perturbation and stronger signature concordance. Meaning: If PZL-26 only inhibits LONP1, then combining PZL-26 with a LONP1 knockdown should not produce much additional effect beyond either perturbation alone as they hit the same node. Increased protein abundance is ambiguous as this can arise from processes unrelated to LONP1 inhibition.

Section: "LONP1 inhibition does not affect TFAM protein levels in 143B"
TFAM, mtDNA, and relevance to probe validation. This section reports that PZL-26 does not alter TFAM levels in 143B cells and causes minimal change in mtDNA copy number, followed by an analysis of a putative N-terminal degron in TFAM. The reader is unclear as to relevance of the degron analysis to validating PZL-26 as a LONP1 probe, especially given the negative TFAM result in 143B. The text also notes prior observations that TFAM is stabilized in rho zero cells treated with bortezomib (used as a LONP1 inhibitor). If rho zero cells provide a sensitive model for detecting LONP1-dependent TFAM turnover, it would make sense to use this system to test the efficacy of PZL-26.
Overall, the mechanistic narrative of this section could be sharpened by more convincingly. demonstrating MMADHC as a selective cellular substrate of LONP1 that can be used as a biomarker for LONP1 inhibition by PZL-26. For TFAM, align cell model choice with prior sensitivity (e.g., rho zero cells) or explicitly temper claims if PZL-26 does not block TFAM turnover.

Section: "LONP1 is required for mitochondrial activity and protein homeostasis".
Txhe results support the notion that PZL-26 induces mitochondrial stress. Suggest tightening the following:
1) The screen was performed under PZL-26 treatment, not under verified cellular LONP1 inhibition. As written, phrases like "under LONP1 inhibition" presume the mechanism. Reframe as "under PZL-26 treatment" unless you provide direct target-engagement and chemical-genetic epistasis.
2) The word "driver" implies causality. In pooled CRISPR, these are sensitizers or fitness modifiers under a specific pressure. Rename to "genes whose loss decreases fitness under PZL-26."
3) Authors acknowledge that proteomics cannot distinguish direct from indirect changes. Direct changes can be addressed by performing substrate-specific kinetics for LONP1-specific substrates, and controls for other protease-specific substrates if known.

Dear Dr Hebbar,

We thank you and the two reviewers for the careful and constructive evaluation of our manuscript. The reviewers have raised a number of important points, and addressing them has substantially strengthened the study. In particular, we have generated new data demonstrating direct cellular target engagement of LONP1 by PZL-26, chemical-genetic concordance with LONP1 knockdown, dose-dependent stabilisation of candidate substrates, and TFAM regulation by PZL-26 in a sensitised cellular model. These results are presented in updated main and expanded view figures: cellular target engagement by CETSA is now incorporated into Fig 4 (panels A and B); the chemical-genetic epistasis data are now incorporated into Fig 3 (panels B–D) with an accompanying heatmap in new EV Fig 4; the orthogonal peptide-level mass spectrometry validation is presented as new EV Fig 3; and the TFAM material, in line with Reviewer 2’s recommendation to temper the TFAM narrative, has been moved to the supplementary section as new EV Fig 5 (which includes the ddC depletion experiment) and new EV Fig 6 (TFAM degron and structural analysis). We have also revised the manuscript text to correct attribution issues, temper conclusions where appropriate, and adopt more precise terminology throughout.

We respond to each reviewer comment below.

Reviewer 1

#1. The reviewer writes: *“Given multiple functions (chaperone activity, protease, and mtDNA maintenance) of LONP1, it would be valuable to discuss which LONP1 activity is inhibited by compound PZL-26.”*

Our response: We thank the reviewer for this helpful suggestion. PZL-26 inhibits the proteolytic activity of LONP1. The cryo-EM density places PZL-26 in the proteolytic active site, and the in vitro inhibition assays measure peptide hydrolysis. We have not directly tested effects on the chaperone or DNA-binding activities of LONP1, and we now state this explicitly in the revised manuscript. The Discussion has been expanded to address this point and to note that the chaperone and DNA-binding functions of LONP1 reside in domains distinct from the proteolytic active site, and are therefore unlikely to be directly affected by PZL-26 binding.

In the discussion, we now write:

PZL-26 inhibits the proteolytic activity of LONP1, but LONP1 also has other reported activities, including a chaperone function and binding to mtDNA. The chaperone and DNA-binding functions reside in domains distinct from the proteolytic active site and are therefore unlikely to be directly engaged by the inhibitor. Consistent with this, siRNA-mediated knockdown of LONP1, which removes all of its activities, produced a proteomic signature closely resembling that of PZL-26 treatment (Fig 3A, B), and co-treatment of LONP1-depleted cells with PZL-26 gave no

further protein accumulation (Fig 3D). The phenotype is thus dominated by loss of proteolytic activity, with no separable contribution from the chaperone or DNA-binding functions detectable at the level of the steady-state proteome, although effects on mtDNA maintenance or nucleoid organisation would not necessarily be captured by proteomics and cannot be formally excluded.The cellular phenotypes described here should accordingly be interpreted as consequences of proteolytic inhibition.

#2. The reviewer writes: “The figure 3 in this manuscript should provide a validation of quantitative proteomics by another biochemical approach, such as Western Blotting.”

Our response: We agree with the reviewer that orthogonal validation of the proteomics data presented in Fig 3 is important. We note that Fig 4C (corresponding to the previous Fig 4A) already provides immunoblot validation for MMADHC, the candidate LONP1 substrate that is most extensively characterised in the manuscript. To extend this orthogonal validation to additional substrates, we have performed targeted peptide-level quantification by mass spectrometry on four further candidates (ECHDC3, NDUFB9, TRUB2 and MRPL13) across a PZL-26 dose range of 20, 100 and 250 nM. The results are presented in new EV Fig 3. Each protein was quantified from between three and eight independent tryptic peptides, providing 21 independent quantifications across the four proteins. All peptides showed concordant, dose-dependent accumulation in response to PZL-26 treatment.

The combination of immunoblot validation for MMADHC (Fig 4C) and targeted peptide-level MS for four further candidates (new EV Fig 3) provides orthogonal support for the proteomics in Fig 3 across five candidate substrates. The new dataset additionally addresses the request of Reviewer 2 for dose-dependent substrate stabilisation in cells.

In the result section, we have added the following text:

Targeted peptide-level mass spectrometry of four candidate substrates (ECHDC3, NDUFB9, TRUB2 and MRPL13) confirmed concordant, dose-dependent accumulation across multiple independent tryptic peptides per protein (EV Fig 3), providing an orthogonal quantitative validation of the proteomic dataset.

#3. The reviewer writes: “The manuscript should offer a clear explanation why there is no change in the level of TFAM while a reduction in mtDNA levels.”

Our response: We thank the reviewer for raising this important point. The unchanged total TFAM levels in 143B cells treated with PZL-26 are in fact the outcome predicted by the established model of Lu et al. (2013), in which DNA-bound TFAM is resistant to LONP1, while free TFAM is rapidly degraded. The modest reduction in mtDNA copy number observed under PZL-26 treatment is likely a secondary consequence of mitochondrial stress caused by LONP1 inhibition rather than a direct effect on TFAM dynamics. Under normal conditions, the free

TFAM that would arise as mtDNA copy number declines would be rapidly degraded by LONP1, leading to a reduction in total TFAM. With LONP1 simultaneously inhibited by PZL-26, this free TFAM is instead stabilised, and total cellular TFAM is therefore maintained at steady state. The unchanged steady-state level we observe is precisely the predicted outcome of LONP1 inhibition in cells that retain a substantial pool of mtDNA. This is analogous to the observation by Lu et al. (Fig 3E of that study) that bortezomib stabilises TFAM in ρ^0 and ρ-low cells, where free TFAM accumulates and is normally degraded by LONP1, but not in ρ^+ cells where TFAM is predominantly DNA-bound and protected.

To test this interpretation directly, we have used ddC to deplete mtDNA prior to PZL-26 treatment (new EV Fig 5B–D). In ddC-treated 143B cells, where mtDNA has been reduced by approximately 70% (EV Fig 5C), PZL-26 produces an upward trend in TFAM levels relative to ddC alone (EV Fig 5B, D; $p = 0.0545$). The magnitude of this effect is in good agreement with the transient and modest stabilisation reported by Lu et al. upon Lon knockdown in mtDNA-deficient cells (Fig 2C, D of that study). We note that the combined treatment with ddC and PZL-26 imposes substantial dual stress on the cells, which may further limit the magnitude of TFAM rescue achievable in this model.

We have revised the result section to present this interpretation, which reconciles our 143B observations with the established Lu et al. model. In line with Reviewer 2's recommendation under point #8, the detailed TFAM and degron material has been moved to the supplementary section (new EV Fig 5 and new EV Fig 6) so that the main text remains focused on probe validation.

We now write:

TFAM is another well-characterized in vitro target of LONP1-mediated degradation. However, treatment with PZL-26 did not result in altered TFAM protein levels in 143B cells (Fig 4C). In addition, we observed a slight decrease in mtDNA levels (EV Fig 5A), likely a downstream consequence of the broader mitochondrial dysfunction caused by LONP1 inhibition, rather than the expected upregulation that would typically accompany increased TFAM levels. Under normal conditions, this reduction in mtDNA would be expected to release free TFAM, which would in turn be degraded by LONP1; with LONP1 inhibited by PZL-26, this free TFAM is instead stabilised, and total TFAM levels are therefore maintained. Previous studies have shown that treatment with bortezomib can lead to stabilization of TFAM in ρ^0 cells lacking mtDNA (Lu et al, 2013). To test whether mtDNA-free TFAM is sensitive to PZL-26 in 143B cells, we depleted mtDNA by ddC treatment prior to addition of PZL-26 (EV Fig 5B). qPCR quantification confirmed that ddC reduced mtDNA copy number by approximately 70% in 143B cells (EV Fig 5C). Under these conditions, PZL-26 produced an upward trend in TFAM levels relative to ddC alone (EV Fig 5B, D; $p = 0.0545$), consistent with the transient and modest stabilisation reported by Lu et

al. (2013) upon Lon knockdown in mtDNA-deficient cells. Combined with our results using a selective LONP1 inhibitor, these findings support a model in which LONP1 selectively degrades surplus TFAM that is not bound to mtDNA.

Reviewer 2

#4. The reviewer writes: *"Cellular target engagement and selectivity needs to be demonstrated. The nanomolar inhibition of purified LONP1 by PZL-26, together with cryo-EM density for PZL-26 in the proteolytic active site, is compelling. The authors are well-positioned to validate PZL-26 as a selective LONP1 inhibitor in cells. Currently, the study lacks direct evidence demonstrating mitochondrial LONP1 engagement under physiological conditions (e.g., in cultured cells). Because off-target effects are common, PZL-26 could also affect other mitochondrial or cellular proteases. Notably, bortezomib has been reported to inhibit LONP1 and additional mitochondrial proteases, insights that informed development of more selective proteasome inhibitors. Cell-based experiments to substantiate on-target action and help to rule out off-target effects could include rescue experiments expressing LONP1 wild-type or variants refractory to PZL-26, or proteomic approaches to identify cellular proteins interacting with PZL-26."*

Our response: We thank the reviewer for this important point. We agree that the original submission did not directly demonstrate cellular target engagement and selectivity, and that this is essential for establishing PZL-26 as a useful chemical probe. We have addressed this concern through three complementary lines of evidence.

The most direct test of on-target action is the chemical-genetic epistasis experiment that the reviewer themselves outlines in Comment 2.4: a true on-target inhibitor should produce no additional effect when combined with a genetic knockdown of the same target, because both perturbations converge on the same node. We have performed precisely this experiment, and the results are now incorporated into Fig 3 (panels B–D) with an accompanying heatmap in new EV Fig 4. The proteins that accumulate upon LONP1 siRNA knockdown (Fig 3B) also accumulate upon combined LONP1 KD and PZL-26 treatment (Fig 3C), and the direct comparison of LONP1 KD + PZL-26 versus LONP1 KD alone shows that PZL-26 does not produce further accumulation of these proteins on top of the genetic perturbation (Fig 3D, new EV Fig 4). The proteome-wide non-additivity is the predicted signature of an on-target inhibitor and argues directly against meaningful contributions from off-target proteases at the concentrations used. Had PZL-26 acted on additional mitochondrial proteases, we would have expected further accumulation of their substrates on top of the LONP1 knockdown, which is not observed.

To complement this functional test of on-target action with direct evidence of binding, we have performed a cellular thermal shift assay (CETSA) in 143B cells. Given its central role in establishing on-target binding, this experiment is now incorporated into the main text as Fig 4A,

B. PZL-26 treatment produces a clear right-shift in the thermal denaturation curve of LONP1 in intact cells, indicating direct binding of the compound to LONP1 within mitochondria under physiological conditions.

Finally, we have extended the dose-response analysis to four candidate LONP1 substrates (ECHDC3, NDUFB9, TRUB2 and MRPL13) using targeted peptide-level mass spectrometry across a PZL-26 dose range of 20, 100 and 250 nM (new EV Fig 3). All four proteins show concordant, dose-dependent accumulation across multiple independent tryptic peptides per protein, consistent with on-target LONP1 inhibition.

Taken together, the chemical-genetic epistasis (Fig 3B–D, new EV Fig 4), direct binding in cells by CETSA (Fig 4A, B), and dose-dependent substrate stabilisation (new EV Fig 3) support PZL-26 as a cellular LONP1 inhibitor with on-target activity at the concentrations used in this study. We have revised the text to reference these new data and to acknowledge that the original biochemical and structural data alone did not establish cellular target engagement.

#5. The reviewer writes: “Accurate attribution of the FRET substrate. The Methods state, ‘A FRET-based assay was developed using the peptide YRGIT(2Abu)SGRQK, end-labeled with the Dabcyl/5-FAM fluorophore/quencher pair,’ implying novelty. However, this peptide substrate FRETN-89-98Abu was reported previously by Lee and colleagues (Fishovitz et al., 2011; Lee and Berdis, 2001). Please revise the text to credit prior work (e.g., ‘we used the previously described FRET substrate …’). This correction is important for proper attribution and for readers to contextualize potency measurements across studies.”

Our response: We thank the reviewer for pointing out this attribution issue. The reviewer is entirely correct. We have revised the Methods section to read: “We used the previously described FRET substrate FRETN-89-98Abu (Lee and Berdis, 2001; Fishovitz et al., 2011), consisting of the peptide YRGIT(2Abu)SGRQK end-labelled with a Dabcyl/5-FAM fluorophore/quencher pair.” Both references have been added to the bibliography. We apologise for the original omission.

#6. The reviewer writes: “MMADHC as a putative LONP1 substrate. The manuscript states: ‘In agreement with published results, MMADHC … has significant accumulation in cells treated with X (Fig. 4A),’ citing Froese et al., 2015. However, Froese et al. do not address LONP1 or MMADHC proteolysis; thus, the citation does not establish prior precedent for MMADHC as a LONP1 substrate. Please either (1) provide a reference that specifically links MMADHC turnover to LONP1, or (2) revise the text to avoid implying prior evidence. In vitro data presented showing degradation of recombinant MMADHC by purified LONP1 supports the possibility that MMADHC is a direct substrate. However as written, the claim that these data ‘reinforce its role as a direct target of LONP1 and a potential biomarker for protease activity’ overstates the

conclusion. Consider tempering to: 'support MMADHC as a candidate LONP1 substrate and a putative biomarker,' pending cellular target-engagement evidence."

Our response: We thank the reviewer for these constructive suggestions. We agree on both points. The Froese et al. citation has been removed from this context, as it does not in fact address MMADHC turnover by LONP1. The text has been revised to read: "MMADHC accumulates in a dose-dependent manner in cells treated with PZL-26, increasing approximately six-fold at 250 nM (Fig 4C), and is degraded by purified LONP1 in a time-dependent manner in vitro (Fig 4D). These data support MMADHC as a candidate LONP1 substrate and a putative biomarker of LONP1 inhibition." We have adopted the wording suggested by the reviewer. The new cellular target engagement data (Fig 3B–D, new EV Fig 3, new EV Fig 4, and Fig 4A, B; see response to point 4) further support this interpretation.

#7. The reviewer writes: *"Limited convergence of PZL-26 inhibition with genetic perturbation. A 55% overlap in protein accumulation profiles between LONP1 siRNA knockdown and PZL-26 treatment (Fig 3C) is suggestive but not decisive. Partial overlap could reflect off-target effects, different perturbation magnitude/kinetics, or secondary effects. True on-target inhibitors typically show non-additive/epistasis with the matching genetic perturbation and stronger signature concordance. Meaning: If PZL-26 only inhibits LONP1, then combining PZL-26 with a LONP1 knockdown should not produce much additional effect beyond either perturbation alone as they hit the same node. Increased protein abundance is ambiguous as this can arise from processes unrelated to LONP1 inhibition."*

Our response: We thank the reviewer for this constructive suggestion. We agree that the 55% overlap reported in the original Fig 3C was suggestive rather than decisive, and that the chemical-genetic epistasis experiment proposed by the reviewer is the more rigorous test. We have performed precisely this experiment, and the results are now incorporated into Fig 3 (panels B–D) with an accompanying heatmap in new EV Fig 4 (also discussed in our response to point 4). The direct comparison of LONP1 KD + PZL-26 versus LONP1 KD alone shows that PZL-26 produces no further accumulation of LONP1-responsive proteins on top of the genetic perturbation (Fig 3D, new EV Fig 4). This non-additivity is the predicted signature of an on-target inhibitor and supports the interpretation that the effects of PZL-26 on the proteome are mediated through LONP1 inhibition.

We note that a small number of proteins do show modest differences between LONP1 KD and LONP1 KD + PZL-26 (visible in Fig 3D). This is expected, as siRNA-mediated knockdown and small-molecule inhibition differ in their kinetics and in the degree of residual LONP1 activity, and the two perturbations are therefore unlikely to be quantitatively identical at the proteome level. The overall pattern is nonetheless one of marked non-additivity, consistent with both

perturbations converging on the same node. The text has been revised to present this interpretation and to refer to the updated Fig 3 and new EV Fig 4.

#8. The reviewer writes: *"TFAM, mtDNA, and relevance to probe validation. This section reports that PZL-26 does not alter TFAM levels in 143B cells and causes minimal change in mtDNA copy number, followed by an analysis of a putative N-terminal degron in TFAM. The reader is unclear as to relevance of the degron analysis to validating PZL-26 as a LONP1 probe, especially given the negative TFAM result in 143B. The text also notes prior observations that TFAM is stabilized in rho zero cells treated with bortezomib (used as a LONP1 inhibitor). If rho zero cells provide a sensitive model for detecting LONP1-dependent TFAM turnover, it would make sense to use this system to test the efficacy of PZL-26. Overall, the mechanistic narrative of this section could be sharpened by more convincingly demonstrating MMADHC as a selective cellular substrate of LONP1 that can be used as a biomarker for LONP1 inhibition by PZL-26. For TFAM, align cell model choice with prior sensitivity (e.g., rho zero cells) or explicitly temper claims if PZL-26 does not block TFAM turnover."*

Our response: We thank the reviewer for this useful suggestion, which has led to a substantial restructuring of the manuscript. We agree that the TFAM material sits awkwardly in a paper whose primary aim is the validation of PZL-26 as a chemical probe, and that MMADHC is the more useful cellular biomarker. We have therefore restructured the manuscript as follows.

The main text now presents a more focused and shorter probe validation narrative, built around three complementary lines of evidence: chemical-genetic epistasis with LONP1 siRNA knockdown (Fig 3B–D, new EV Fig 4), direct cellular target engagement by CETSA (Fig 4A, B, moved to the main text given its central role in establishing on-target binding), and dose-dependent accumulation of LONP1 substrates including MMADHC (Fig 4C, D) and four further candidates (new EV Fig 3). MMADHC is now presented as the primary cellular biomarker of LONP1 inhibition by PZL-26, supported by dose-dependent accumulation in cells (Fig 4C), time-dependent degradation by purified LONP1 in vitro (Fig 4D), and concordant dose-response across multiple independent substrates (new EV Fig 3).

In line with the reviewer's recommendation to temper the TFAM narrative, the TFAM material and the N-terminal degron analysis have been moved to the supplementary section (new EV Fig 5 and new EV Fig 6). We have chosen to retain these data in supplementary form because the regulation of TFAM by LONP1 remains an active topic of discussion in the mitochondrial literature, and our observations contribute to that discussion. We make explicit in the revised text that TFAM is not used as a biomarker in this study, but is examined as a previously proposed LONP1 substrate. New EV Fig 5 shows that in ddC-treated 143B cells, where mtDNA has been reduced by approximately 70%, PZL-26 produces an upward trend in TFAM levels relative to ddC alone ($p = 0.0545$), in agreement with the transient and modest stabilisation

reported by Lu et al. (2013) upon Lon knockdown in mtDNA-deficient cells. The unchanged total TFAM levels in 143B cells with intact mtDNA are also consistent with the model of Lu et al., in which DNA-bound TFAM is resistant to LONP1 while free TFAM is rapidly degraded (see our response to point 3 for the detailed mechanistic interpretation). The degron analysis and structural interpretation that follow in new EV Fig 6 provide the mechanistic basis for this selectivity.

We chose ddC-mediated mtDNA depletion rather than ρ^0 cells for this purpose. ddC provides a controllable, partial depletion of mtDNA in the same 143B background used throughout the manuscript, avoiding the irreversible nuclear-encoded adaptations that accumulate in ρ^0 lines and allowing direct comparison with the other data in the study.

#9. The reviewer writes: *"LONP1 is required for mitochondrial activity and protein homeostasis. The results support the notion that PZL-26 induces mitochondrial stress. Suggest tightening the following: 1) The screen was performed under PZL-26 treatment, not under verified cellular LONP1 inhibition. As written, phrases like 'under LONP1 inhibition' presume the mechanism. Reframe as 'under PZL-26 treatment' unless you provide direct target-engagement and chemical-genetic epistasis. 2) The word 'driver' implies causality. In pooled CRISPR, these are sensitizers or fitness modifiers under a specific pressure. Rename to 'genes whose loss decreases fitness under PZL-26.' 3) Authors acknowledge that proteomics cannot distinguish direct from indirect changes. Direct changes can be addressed by performing substrate-specific kinetics for LONP1-specific substrates, and controls for other protease-specific substrates if known."*

Our response: We thank the reviewer for these constructive suggestions and have adopted them throughout the manuscript.

(i). We have replaced "under LONP1 inhibition" with "under PZL-26 treatment" throughout the cellular sections, except in passages where target engagement is now independently established by the new CETSA and epistasis data (Fig 3B–D, new EV Fig 4, and Fig 4A, B; see response to point 4). In those passages the mechanistic statement is warranted, and we have made the supporting evidence explicit in the text.

(ii). The term "drivers" has been replaced in the CRISPR screen section. We now describe these as "genes whose loss decreases fitness under PZL-26 treatment," following the reviewer's suggested wording, and analogous language has been used elsewhere in the screen-related text.

(iii). We agree that the proteomics data alone cannot distinguish direct LONP1 substrates from proteins that accumulate as an indirect consequence of LONP1 inhibition, and we now state this explicitly in the Discussion. We note that the new chemical-genetic epistasis experiment (Fig 3B–D, new EV Fig 4; see response to point 4) addresses a related but separate question:

whether the proteome-wide response to PZL-26 is mediated through LONP1 at all. The non-additivity between PZL-26 and LONP1 knockdown shows that it is, and that off-target proteases do not make a meaningful contribution at the concentrations used. The question of which individual accumulating proteins are direct LONP1 substrates is distinct. We have selected MMADHC as our primary in vitro-validated cellular substrate, supported by both time-dependent degradation by purified LONP1 (Fig 4D) and dose-dependent accumulation in cells (Fig 4C). The four additional substrates presented in new EV Fig 3 (ECHDC3, NDUFB9, TRUB2 and MRPL13) serve as an independent dose-response validation of the cellular proteomics rather than as fully characterised direct substrates, and we now describe them as candidate substrates in the revised text. The reviewer's suggestion of controls using specific substrates of other mitochondrial proteases is well taken; however, validated specific substrates of ClpXP and AFG3L2 are not as well established in the literature as those of LONP1, which limits the practical application of such controls at present. The Discussion has been updated to make these distinctions explicit.

July 21, 2026

RE: Life Science Alliance Manuscript #LSA-2025-03535-TR

Prof. Claes M Gustafsson
University of Gothenburg
Department of Medical Biochemistry and Cell Biology
P.O. Box 440
Gothenburg 405 30
Sweden

Dear Dr. Gustafsson,

Thank you for submitting your revised manuscript entitled "A Selective Inhibitor Reveals Roles for LONP1 in Mitochondrial Proteostasis and Cellular Metabolism".

Your manuscript was evaluated by both original reviewers whose comments are included below. As you will note, Reviewer 2 comments that your revised manuscript has addressed many of the previous concerns. This reviewer has also specified pending concerns that we agree must be resolved in the ms text. These are related to (1) improving the accuracy of descriptions (comments paragraphs 1-3), and, (2) toning down claim related to metabolism throughout the ms text and title.
Reviewer 2 suggests a citation (Lu et al)-related change in the description of the discussion/introduction of TFAM. If you do not make this change in the introduction/discussion, please include your rationale for the same in a point-by-point response letter.

In line with the reviewers' overall evaluation, we would be happy to publish your paper in Life Science Alliance pending final revisions necessary to meet our formatting guidelines, and resolution of concerns from Reviewer 2 as described above. Please include a point-by-point response to these concerns.

MANUSCRIPT ORGANIZATION AND FORMATTING:

To avoid unnecessary delays in the acceptance and publication of your paper, please read the following information carefully. Full guidelines are available on our Instructions for Authors page, https://www.life-science-alliance.org/authors

Submission of a paper that does not conform to Life Science Alliance guidelines will delay the acceptance of your manuscript.
-Please integrate the text under heading, 'EXPANDED VIEW TEXT' that describes some of the supplementary figures into the main ms text.
-Please include information on guide sequences for the CRISPR-Cas9 screening.
-Please provide details on the LONP1 antibody (immunoblotting, line 408) and the generation of the homemade TFAM antibody mentioned on line 393 or provide a citation if its generation is previously described. Related to this, please provide concentrations of all antibodies used in this work.
-Kindly provide a source for the inhibitors used in this work (PZL-26 or bortezomib, line 354).
-Information on primers used for qPCR must be included in the methods section.
-LSA allows supplementary figures, but no EV Figures; please update your callouts for the Supplementary Figures in the manuscript Fig EV1A=Fig S1A; while supplementary figures use the system supplementary Fig S1.
-Please include molecular weight markers for blots in Figure 4, Supplemental Figure 5.
-Please remind your co-corresponding authors to add ORCID IDs to their accounts - they should have received instructions on how to do so .
-Please add the X and Bluesky handles of your host institute/organization, as well as your own, and/or one of the authors, in our system.
-Please rename "DISCLOSURE AND COMPETING INTERESTS STATEMENT" to "Conflict of Interest".
-The "Data Availability" section should be placed after the Materials & Methods section. This would especially relate to the mass spec data and structural data. Please consult our guidelines at https://www.life-science-alliance.org/manuscript-prep#format.
-Please add an Author Contributions section to your main manuscript text.
-Please add callouts for Figures S5B-D and S6A-D to your main manuscript text.
-Please be sure that the authorship listing and order is correct

We encourage our authors to provide original source data, particularly uncropped/-processed electrophoretic blots and spreadsheets for the main figures of the manuscript. If you would like to add source data, we would welcome one PDF/Excel-file per figure for this information. These files will be linked as supplementary "Source Data" files.

It is Life Science Alliance policy that if requested, original data images must be made available to the editors. Failure to provide original images upon request will result in unavoidable delays in publication. Please ensure that you have access to all original data images prior to final submission.

If you are planning a press release on your work, please inform us immediately to allow informing our production team and scheduling a release date.

We welcome submissions of potential cover images for the issue of LSA in which your work would appear. If you have high quality images associated with this work, please feel free to email these, with a caption, to the journal office.

LSA encourages authors to provide a 30-60 second video where the study is briefly explained. These videos will be appear embedded with the manuscript online at Life Science Alliance, and on social media to promote the published paper and authors (for examples, see https://docs.google.com/document/d/1-UWCfbE4pGcDdcgzcmiuJl2XMBJnxKYeqRvLLrLSo8s/edit?usp=sharing). Corresponding or first-authors are welcome to submit the video. Please submit only one video per manuscript. The video can be emailed to contact@life-science-alliance.org

FINAL FILES:

To upload the final version of your manuscript, please log in to your account: https://lsa.msubmit.net/cgi-bin/main.plex
You will be guided to complete the submission of your revised manuscript and to fill in all necessary information. Please get in touch in case you do not know or remember your login name.

The following items are required for acceptance.

-- An editable version of the final text (.DOC or .DOCX) is needed for copyediting (no PDFs).

-- High-resolution figure, supplementary figure and video files uploaded as individual files: See our detailed guidelines for preparing your production-ready images, https://www.life-science-alliance.org/authors

-- Summary blurb (enter in submission system): A short text summarizing in a single sentence the study (max. 200 characters including spaces). This text is used in conjunction with the titles of papers, hence should be informative and complementary to the title. It should describe the context and significance of the findings for a general readership; it should be written in the present tense and refer to the work in the third person. Author names should not be mentioned.

The license to publish form must be signed before your manuscript can be sent to production. A link to the license to publish form will be available to the corresponding author only. Please take a moment to check your funder requirements.

Reviews, decision letters, and point-by-point responses associated with peer-review at Life Science Alliance will be published, alongside the manuscript. If you do want to opt out of having the reviewer reports and your point-by-point responses displayed, please let us know immediately.

Thank you for your attention to these final processing requirements. Please revise and format the manuscript and upload materials as soon as you are able.

Thank you for this interesting contribution to the literature. We look forward to publishing your paper in Life Science Alliance.

Sincerely,

Sarita Hebbar, PhD
Scientific Editor
Life Science Alliance
http://www.lsajournal.org

Reviewer #1 (Comments to the Authors (Required)):

I have no further comments on this revised manuscript, thanks!

Reviewer #2 (Comments to the Authors (Required)):

The resubmitted manuscript by Szilagyi et al. is substantially stronger with respect to its principal objective of establishing PZL-26 as a cellular inhibitor of LONP1. The authors responded constructively to the major concerns in the initial review by adding

cellular target-engagement data, a chemical-genetic epistasis experiment, dose-dependent analysis of candidate proteins, and a more appropriately qualified description of MMADHC. They also moved most of the TFAM degradation and degron analysis to the Expanded View, which improves the focus of the main manuscript. These changes address several important deficiencies in the original submission.

However, several claims remain broader than the data support, particularly the descriptions of peptide-level validation, inhibitor specificity, cellular metabolism, and the biological interpretation of LONP1-dependent TFAM degradation. Please see the comments below.

Targeted peptide-level MS is not orthogonal validation. The rebuttal describes peptide-level quantification of ECHDC3, NDUFB9, TRUB2, and MRPL13 as "orthogonal quantitative validation" of the proteomic dataset. According to the revised Methods, however, these peptide measurements were extracted from the same TMT mass-spectrometry dataset. Concordant behavior of several uniquely mapped peptides provides useful internal support for the protein-level quantification and helps show that an apparent change is not driven by a single peptide. However, this is not an orthogonal validation because it uses the same samples and analytical platform. The MMADHC immunoblot is the genuinely orthogonal validation presented. More accurate wording for the four additional proteins would be "independent peptide-level support within the proteomic dataset" or "peptide-level confirmation of the protein-level quantification."

The revised manuscript correctly acknowledges that proteomics cannot distinguish direct LONP1 substrates from proteins that accumulate indirectly. ECHDC3, NDUFB9, TRUB2, and MRPL13 should therefore be described consistently as candidate substrates or LONP1-responsive proteins in the Results, figure legends, and Discussion unless direct degradation by LONP1 is demonstrated.

"Specific" versus "selective" cellular inhibitor. CETSA, chemical-genetic non-additivity with LONP1 knockdown, and the enrichment of mitochondrial proteins collectively provide strong evidence that PZL-26 engages LONP1 in cells and that the measured proteomic response is predominantly on target. Nevertheless, these experiments do not establish that PZL-26 has no other cellular targets. Therefore, it would be more accurate to describe PZL-26 as "a potent, highly selective cellular inhibitor of LONP1" rather than as a "specific inhibitor."

This distinction is important because PZL-26 is proposed as a chemical probe. A comprehensive claim of cellular specificity would generally require a broader selectivity profile and ideally a matched inactive control, an inhibitor-resistant LONP1 rescue, or another experiment capable of detecting pharmacology outside the measured proteomic response. The new data strongly support on-target activity but do not exclude all off-target effects.

Claims regarding cellular metabolism are overstated. Although the revised manuscript substantially strengthens the validation of PZL-26 as an on-target cellular inhibitor of LONP1, the presented results don't directly establish roles for LONP1 in "cellular metabolism." The study measures changes in protein abundance, pathway enrichment, and genetic fitness dependencies involving OXPHOS and mitochondrial gene-expression pathways. These findings demonstrate that LONP1 proteolytic activity is important for mitochondrial proteostasis and for the cellular maintenance or tolerance of respiratory-chain components. They do not directly measure metabolic activity or flux.

In the absence of direct measurements such as oxygen consumption, ATP production, respiratory-complex activity, metabolite abundance, substrate utilization, redox state, or isotope-traced flux, the title and conclusions should emphasize mitochondrial proteostasis, mitochondrial gene expression, and OXPHOS dependencies rather than "cellular metabolism."

Recommend revising the title, Abstract, and conclusions to focus on mitochondrial proteostasis, OXPHOS-complex homeostasis, and mitochondrial gene expression rather than cellular metabolism.
Revised titles might be:
"A Selective Inhibitor Reveals Roles for LONP1 in Mitochondrial Proteostasis and Gene Expression"

"A Selective LONP1 Inhibitor Reveals Mitochondrial Proteostasis and OXPHOS Dependencies"

The interpretation of TFAM degradation remains incomplete. In the Introduction (lines 67-68), the authors characterize LONP1-dependent degradation of TFAM principally as clearance of "surplus" TFAM referring to recent studies from 2018 and 2020 suggesting that LONP1 "primarily degrades excess unbound TFAM, potentially serving a housekeeping role ..." However, later in the manuscript (lines 186-187) the authors refer to a study by Lu et al. from 2013 as an "established model that LONP1 selectively targets DNA-free TFAM". This study showed that TFAM is degraded by LONP1 when mtDNA is absent or markedly depleted, whereas DNA-bound TFAM is protected. They also showed that TFAM variants with mutations that disrupt the DNA-binding activity of HMG box 1 and/or HMG box 2 are degraded in cells that retain mtDNA.

In a distinct set of experiments, phosphorylation of serine residues within HMG box 1 reduced TFAM binding to DNA and promoted rapid LONP1-dependent degradation. Thus, phosphorylation was demonstrated for HMG box 1, whereas site-directed mutagenesis was used to disrupt the DNA-binding functions of HMG box 1 and/or HMG box 2.

This distinction is important as DNA-free TFAM need not be merely a stoichiometric surplus of TFAM over available mtDNA. It can also be generated by a regulated post-translational modification that changes TFAM DNA binding. Referring to the substrate only as "surplus TFAM" narrows the prior model to passive housekeeping and obscures the possibility that LONP1 participates in regulated control of TFAM function and abundance.

The manuscript should be revised to adequately acknowledge the phosphorylation-dependent mechanism emphasized in the study by Lu et al. This omission is especially notable because the authors' own proposed degron overlaps or extends into HMG box 1, the region in which phosphorylation can impair DNA binding. Their structural argument-that folding upon DNA binding shields the degron-is potentially complementary to Lu et al., but the manuscript presents it largely as a passive distinction between bound and unbound conformations.

From my reading, Lu et al. did not propose that LONP1-mediated TFAM degradation ordinarily produces a simple reduction in mtDNA copy number. In mtDNA-depleted rho-low cells, reducing LONP1 stabilized TFAM and was accompanied by increased mtDNA, but the broader conclusion as written is that phosphorylation and proteolysis can rapidly fine-tune TFAM function and abundance that are important for mtDNA maintenance and expression.

Recommended revisions to the TFAM discussion
• replace "surplus" or "excess TFAM" with the mechanistically neutral term "DNA-free TFAM";
• state that DNA-free TFAM may arise from mtDNA depletion, loss of intrinsic HMG-box DNA-binding activity, or phosphorylation-dependent reduction of DNA binding;
• distinguish clearly between phosphorylation of HMG box 1 and mutational disruption of HMG box 1 and/or HMG box 2;
• acknowledge that selective degradation of DNA-free TFAM may constitute both protein quality control and regulated fine-tuning of TFAM function.

RESPONSE TO THE EDITOR AND REVIEWERS

Manuscript ID: LSA-2025-03535-TR

Title: *A Selective Inhibitor Reveals Roles for LONP1 in Mitochondrial Proteostasis and OXPHOS Dependencies*

Dear Dr. Hebbar,

We would like to thank you and the reviewers for the careful evaluation of our revised manuscript and for the constructive comments. We greatly appreciate the time and effort invested in reviewing our work.

We are grateful that Reviewer #1 considers all previous concerns satisfactorily addressed and that Reviewer #2 found the revised manuscript substantially strengthened. We have carefully considered all remaining comments and have revised the manuscript accordingly.

In response to Reviewer #2, we have revised the manuscript to improve the accuracy of several descriptions, replaced the term *specific* with *selective* where appropriate, revised the title and text to better reflect the scope of our data, and updated the discussion of TFAM biology in accordance with the reviewer's suggestions.

In addition, we have addressed all editorial requests. These revisions include incorporation of the Expanded View text into the main manuscript, addition of CRISPR library information, antibody details and antibody dilutions, inhibitor sources, qPCR primer sequences, molecular weight markers, Data Availability, Author Contributions, updated supplementary figure callouts, and all requested formatting changes.

We believe that these revisions have further strengthened the manuscript and hope that it is now suitable for publication in *Life Science Alliance*.

Sincerely,

Claes M. Gustafsson

on behalf of all authors

Response to Reviewer #1

We thank Reviewer #1 for the positive evaluation of our revised manuscript and for confirming that the previous concerns have been satisfactorily addressed. We appreciate the reviewer's careful assessment of our work.

Response to Reviewer #2

We sincerely thank Reviewer #2 for the thorough and thoughtful evaluation of our revised manuscript. We are grateful that the reviewer found the manuscript substantially strengthened and that the additional experiments successfully addressed the major concerns raised during the previous round of review.

Below we address each of the remaining comments in detail.

Comment 1

Targeted peptide-level MS is not orthogonal validation. The rebuttal describes peptide-level quantification of ECHDC3, NDUFB9, TRUB2, and MRPL13 as "orthogonal quantitative validation" of the proteomic dataset. According to the revised Methods, however, these peptide measurements were extracted from the same TMT mass-spectrometry dataset... More accurate wording would be "independent peptide-level support within the proteomic dataset" or "peptide-level confirmation of the protein-level quantification."

Response
We thank the reviewer for this important observation and agree that the peptide-level analyses were derived from the same TMT proteomic dataset and therefore should not be described as orthogonal validation.

Accordingly, we have revised the manuscript throughout. The term "orthogonal validation" has been removed from the Results, Methods, and Supplementary Figure S2 legend and replaced with wording stating that these analyses provide peptide-level support for the protein-level quantification within the same proteomic dataset. We believe that this wording more accurately reflects the experimental design while preserving the value of the peptide-level analyses as internal confirmation of the protein-level measurements.

The corresponding changes have been made in the Results, Methods, and Supplementary Figure S2 legend.

Comment 2

The revised manuscript correctly acknowledges that proteomics cannot distinguish direct LONP1 substrates from proteins that accumulate indirectly. ECHDC3, NDUFB9, TRUB2, and MRPL13 should therefore be described consistently as candidate substrates or LONP1-responsive proteins throughout the manuscript.

Response
We appreciate this suggestion and agree with the reviewer.

The manuscript has been revised throughout to consistently describe ECHDC3, NDUFB9, TRUB2, and MRPL13 as “candidate LONP1 substrates” or “LONP1-responsive proteins” pending direct experimental validation. In contrast, MMADHC is now consistently described as a “direct LONP1 substrate”, supported by both dose-dependent accumulation in cells and direct degradation by purified recombinant LONP1 in vitro.

These revisions have been implemented in the Results, Discussion, Methods, and relevant figure legends to ensure consistent terminology throughout the manuscript.

Comment 3

"Specific" versus "selective" cellular inhibitor. It would be more accurate to describe PZL-26 as "a potent, highly selective cellular inhibitor of LONP1" rather than as a "specific inhibitor."

Response

We agree with the reviewer and have revised the terminology throughout the manuscript. The term "specific" has been replaced with "selective" wherever appropriate, including the title, Abstract, Results, Discussion, and figure legends. We agree that "selective" is the more accurate term: our data define a quantifiable selectivity window (an IC_{50} of 27 nM against LONP1 versus 7.3 µM against the 20S proteasome, an approximately 1000-fold difference), whereas "specific" would imply an absence of activity against all other targets that we have not established and that is difficult to demonstrate for a covalent small-molecule inhibitor.

The revised terminology therefore reflects the experimental evidence while avoiding overstatement of the selectivity profile.

The corresponding changes have been implemented throughout the manuscript.

Comment 4

Claims regarding cellular metabolism are overstated. Although the revised manuscript substantially strengthens the validation of PZL-26 as an on-target cellular inhibitor of LONP1, the presented results do not directly establish roles for LONP1 in "cellular metabolism." The study measures changes in protein abundance, pathway enrichment, and genetic fitness dependencies involving OXPHOS and mitochondrial gene-expression pathways... The title and conclusions should emphasize mitochondrial proteostasis, mitochondrial gene expression, and OXPHOS dependencies rather than "cellular metabolism."

Response

We thank the reviewer for this thoughtful and constructive comment and agree that the conclusions should closely reflect the scope of the presented data.

We agree that our study examines changes in protein abundance, pathway enrichment, and genetic dependencies rather than direct measurements of metabolic activity or metabolic flux.

Accordingly, we have revised the title, Abstract, Results, Discussion, and Conclusions to avoid statements implying direct measurements of cellular metabolism. The revised manuscript now consistently emphasizes mitochondrial proteostasis, mitochondrial gene expression, and oxidative phosphorylation-related pathways, which are directly supported by our proteomic and CRISPR-Cas9 analyses.

The title has been revised from "A Selective Inhibitor Reveals Roles for LONP1 in Mitochondrial Proteostasis and Cellular Metabolism" to "A Selective Inhibitor Reveals Roles for LONP1 in Mitochondrial Proteostasis and OXPHOS Dependencies" to emphasise the mitochondrial pathways directly supported by our proteomic and CRISPR-Cas9 analyses.

Comment 5

The interpretation of TFAM degradation remains incomplete. In the Introduction (lines 67-68), the authors characterize LONP1-dependent degradation of TFAM principally as clearance of "surplus" TFAM referring to recent studies from 2018 and 2020 suggesting that LONP1 "primarily degrades excess unbound TFAM, potentially serving a housekeeping role ..." However, later in the manuscript (lines 186-187) the authors refer to a study by Lu et al. from 2013 as an "established model that LONP1 selectively targets DNA-free TFAM". This study showed that TFAM is degraded by LONP1 when mtDNA is absent or markedly depleted, whereas DNA-bound TFAM is protected. They also showed that TFAM variants with mutations that disrupt the DNA-binding activity of HMG box 1 and/or HMG box 2 are degraded in cells that retain mtDNA.
In a distinct set of experiments, phosphorylation of serine residues within HMG box 1 reduced TFAM binding to DNA and promoted rapid LONP1-dependent degradation. Thus, phosphorylation was demonstrated for HMG box 1, whereas site-directed mutagenesis was used to disrupt the DNA-binding functions of HMG box 1 and/or HMG box 2.

This distinction is important as DNA-free TFAM need not be merely a stoichiometric surplus of TFAM over available mtDNA. It can also be generated by a regulated post-translational modification that changes TFAM DNA binding. Referring to the substrate only as "surplus TFAM" narrows the prior model to passive housekeeping and obscures the possibility that LONP1 participates in regulated control of TFAM function and abundance.

The manuscript should be revised to adequately acknowledge the phosphorylation-dependent mechanism emphasized in the study by Lu et al. This omission is especially notable because the authors' own proposed degron overlaps or extends into HMG box 1, the region in which phosphorylation can impair DNA binding. Their structural argument-that folding upon DNA binding shields the degron-is potentially complementary to Lu et al., but the manuscript presents it largely as a passive distinction between bound and unbound conformations.

From my reading, Lu et al. did not propose that LONP1-mediated TFAM degradation ordinarily produces a simple reduction in mtDNA copy number. In mtDNA-depleted rho-low cells, reducing LONP1 stabilized TFAM and was accompanied by increased mtDNA, but the broader conclusion as written is that phosphorylation and proteolysis can rapidly fine-tune TFAM function and abundance that are important for mtDNA maintenance and expression.

Response
We thank the reviewer for this careful and insightful analysis. We agree that our previous presentation was too narrowly focused on the degradation of TFAM present in excess over available mtDNA and did not adequately acknowledge the broader mechanistic model described by Lu et al.

We have therefore revised the Introduction, Results, and TFAM discussion to distinguish more clearly between the mechanisms that generate DNA-free TFAM and the subsequent susceptibility of DNA-free TFAM to LONP1-mediated degradation. In particular, we now acknowledge that DNA-free TFAM need not arise solely from a stoichiometric excess of TFAM over mtDNA, but may also result from impaired DNA-binding activity or from regulatory mechanisms that reduce TFAM-DNA association.

We have also expanded the discussion of Lu et al. to distinguish explicitly between their phosphorylation experiments involving HMG box 1 and the separate mutational experiments disrupting the DNA-binding functions of HMG box 1 and/or HMG box 2. The revised text describes the phosphorylation-dependent mechanism as one possible means by which TFAM may enter a DNA-free state that is susceptible to LONP1-mediated degradation. Importantly, we now highlight the connection between this previous work and the degron-containing region identified in our study. The degron identified here spans TFAM residues 43-60 and extends into HMG box 1, overlapping the region implicated in phosphorylation-dependent regulation of TFAM DNA binding. We have revised the discussion to emphasize that these observations are potentially complementary. Our data suggest that DNA binding structurally protects the degron-containing region, whereas mechanisms that reduce TFAM-DNA association may increase its accessibility to LONP1. We have phrased this as a possible structural explanation for the preferential degradation of DNA-free TFAM rather than as a demonstrated mechanism for how DNA-free TFAM is generated physiologically.

Finally, we have revised the biological interpretation so that LONP1-mediated TFAM degradation is no longer presented as a simple mechanism for reducing mtDNA copy number or solely as passive housekeeping. The revised text now acknowledges that preferential degradation of DNA-free TFAM may contribute both to mitochondrial protein quality control and to regulated fine-tuning of TFAM abundance and function in relation to mtDNA maintenance and expression.

Comment 6

replace "surplus" or "excess TFAM" with the mechanistically neutral term "DNA-free TFAM";

Response
We agree with the reviewer and have revised the manuscript accordingly. Throughout the revised manuscript, we now use the mechanistically neutral term “DNA-free TFAM” rather than “surplus TFAM” or “excess TFAM” when referring to the pool of TFAM that is susceptible to LONP1-mediated degradation.
We have also removed wording that characterized this process principally as clearance of excess TFAM or as a passive housekeeping mechanism. This revised terminology more accurately reflects that the relevant feature of the substrate is its DNA-free state rather than the mechanism by which that state was generated.

Comment 7

state that DNA-free TFAM may arise from mtDNA depletion, loss of intrinsic HMG-box DNA-binding activity, or phosphorylation-dependent reduction of DNA binding;

Response
We agree and have expanded the text accordingly. The revised manuscript now explicitly states that DNA-free TFAM may arise through multiple mechanisms, including mtDNA depletion, loss or impairment of intrinsic HMG-box DNA-binding activity, and phosphorylation-dependent reduction of TFAM DNA binding.
This revision separates the origin of the DNA-free TFAM pool from its subsequent recognition and degradation by LONP1 and provides a more complete description of the model supported by previous work.

Comment 8

distinguish clearly between phosphorylation of HMG box 1 and mutational disruption of HMG box 1 and/or HMG box 2;

Response
We thank the reviewer for pointing out this important distinction. The revised manuscript now clearly separates these two sets of experiments described by Lu et al.
We now state that phosphorylation of serine residues within HMG box 1 was associated with reduced TFAM DNA binding and enhanced LONP1-dependent degradation. We separately describe the mutational experiments in which disruption of DNA-binding functions in HMG box 1 and/or HMG box 2 rendered TFAM susceptible to degradation in cells retaining mtDNA.
This distinction clarifies that the mutational experiments demonstrate the consequences of impaired TFAM DNA binding, whereas the phosphorylation experiments provide a possible regulatory mechanism by which TFAM DNA binding and its susceptibility to LONP1-mediated degradation may be altered.

Comment 9

acknowledge that selective degradation of DNA-free TFAM may constitute both protein quality control and regulated fine-tuning of TFAM function.

Response
We agree with the reviewer and have revised the Introduction and TFAM discussion accordingly. The revised manuscript now acknowledges that preferential degradation of DNA-free TFAM may contribute both to mitochondrial protein quality control and to regulated fine-tuning of TFAM abundance and function.
We have also revised the text to avoid implying that LONP1-dependent degradation of TFAM normally serves simply to reduce mtDNA copy number. Instead, we now describe a broader model in which changes in TFAM DNA binding can alter the fraction of TFAM accessible to LONP1, thereby providing a potential mechanism for adjusting TFAM turnover in relation to mtDNA maintenance and expression.
These revisions provide a more balanced interpretation of the previous literature and integrate the phosphorylation-dependent mechanism described by Lu et al. with our finding that the

LONP1-sensitive degron-containing region overlaps HMG box 1 and may be protected when TFAM is bound to DNA.

Concluding remark
We thank the reviewer once again for the thoughtful and constructive comments. We believe that the revisions made in response to these suggestions have improved the clarity, accuracy, and overall presentation of the manuscript while preserving its principal conclusions.

Response to Editorial and Formatting Requests

We thank the Editor for the detailed editorial suggestions. All requested editorial and formatting revisions have been incorporated into the revised manuscript, including:

- Integration of the Expanded View text into the main manuscript.
- Addition of details on the genome-wide Brunello sgRNA library, including the number of sgRNAs, the number of guides per gene, and the non-targeting control sgRNAs.
- Addition of antibody details and antibody dilutions.
- Addition of inhibitor sources.
- Addition of qPCR primer sequences.
- Replacement of EV figure citations with Supplementary Figure citations.
- Addition of molecular weight markers to the requested immunoblots.
- Addition of the Author Contributions section.
- Addition of the Data and code availability section, including deposition information for the structural data (PDB 31SY; EMD-58651), proteomics data (MassIVE MSV000102843), and CRISPR-Cas9 screening data (GEO GSE343387).
- Addition of Supplementary Figure callouts throughout the manuscript.
- Renaming of the Disclosure and Competing Interests Statement to Conflict of Interest.
- Updating the manuscript to conform to the Life Science Alliance formatting requirements.

Final statement

We sincerely thank the Editor and both Reviewers for their careful evaluation of our work and for their constructive suggestions. We believe that the revisions have substantially strengthened the manuscript and improved both its clarity and scientific accuracy.

We hope that the revised manuscript is now suitable for publication in *Life Science Alliance*.

September 9, 2026

RE: Life Science Alliance Manuscript #LSA-2025-03535-TRR

Prof. Claes M Gustafsson
University of Gothenburg
Department of Medical Biochemistry and Cell Biology
P.O. Box 440
Gothenburg 405 30
Sweden

Dear Dr. Gustafsson,

Thank you for submitting your Research Article entitled "A Selective Inhibitor Reveals Roles for LONP1 in Mitochondrial Proteostasis and Cellular Metabolism". It is a pleasure to let you know that your manuscript is now accepted for publication in Life Science Alliance. Congratulations on this interesting work.

The final published version of your manuscript will be deposited by us to PubMed Central upon publication.

Your manuscript will now progress through copyediting and proofing. At the proofing stage, we request you to confirm the accuracy of the citation provided for TFAM antibody details in the 'Materials and Methods' section (page 15).

It is journal policy that authors provide original data upon request.

Your article will publish open access upon publication under a CC-BY license.

Reviews, decision letters, and point-by-point responses associated with peer-review at Life Science Alliance will be published, alongside the manuscript. If you do want to opt out of having the reviewer reports and your point-by-point responses displayed, please let us know immediately.

IMPORTANT: If you will be unreachable at any time, please provide us with the email address of an alternate author. Failure to respond to routine queries may lead to unavoidable delays in publication.

Scheduling details will be available from our production department. You will receive proofs shortly before the publication date. Only essential corrections can be made at the proof stage so if there are any minor final changes you wish to make to the manuscript, please let the journal office know now.

DISTRIBUTION OF MATERIALS:
Authors are required to distribute freely any materials used in experiments published in Life Science Alliance. Authors are encouraged to deposit materials used in their studies to the appropriate repositories for distribution to researchers.

You can contact the journal office with any questions, contact@life-science-alliance.org

Again, congratulations on a very nice paper. I hope you found the review process to be constructive and are pleased with how the manuscript was handled editorially. We look forward to future exciting submissions from your lab.

Sincerely,

Sarita Hebbar, PhD
Scientific Editor
Life Science Alliance
http://www.lsajournal.org
